# Activity profiles and their associations with transitions between fall and dementia: findings from a multistate time-to-event analysis

**DOI:** 10.1186/s13195-026-02140-2

**Published:** 2026-07-10

**Authors:** Benjamin Kröger, Maria M. Ekblom, Hui-Xin Wang, Charlotta Thunborg, Miia Kivipelto, Rui Wang

**Affiliations:** 1 Department of Physical Activity and Health, the Swedish School of Sport and Health Science, GIH, Stockholm, Sweden; 2 Division of Clinical Geriatrics, Department of Neurobiology, Care Sciences and Society, Karolinska Institutet, Karolinska Vägen 37 A, QA32, 171 64 Stockholm, Solna Sweden; 3 National Graduate School On Ageing and Health (SWEAH), Lund, Sweden; 4 Division of Physiotherapy, Department of Neurobiology, Care Sciences and Society, Karolinska Institutet, Stockholm, Sweden; 5 Division of Psychobiology and Epidemiology, Department of Psychology, Stockholm University, Stockholm, Sweden; 6 Department of Clinical Neuroscience, Karolinska Institutet, Stockholm, Sweden; 7 Department of Caring Sciences, Faculty of Health and Occupational Studies, University of Gävle, Gävle, Sweden; 8 Theme Inflammation and Aging, Karolinska University Hospital, Stockholm, Sweden; 9 Institute of Public Health and Clinical Nutrition, University of Eastern Finland, Kuopio, Finland; 10 Ageing Epidemiology Research Unit (AGE), School of Public Health, Faculty of Medicine, Imperial College London, London, UK; 11 Wisconsin Alzheimer’s Disease Research Center, School of Medicine and Public Health, University of Wisconsin, Madison, WI USA

**Keywords:** Dementia, Fall, Latent Class Analysis, Activity Profiles, UK biobank

## Abstract

**Background:**

The potential link between data-driven classifications of individuals’ activity profiles and the occurrence of fall and dementia, including within-person transitions from dementia- or fall-only states to their co-occurrence over time, remains unclear.

**Method:**

To examine whether distinct activity profiles were associated with the risk of fall, dementia, and their transitions over time in middle-aged and older adults, this prospective longitudinal study included 288,287 adults (53.6% women) aged 40–69 years at baseline (2006–2010) from the UK Biobank. Latent class analysis was used to classify participants into distinct activity profiles based on self-reported physical activity, transportation, work, social, and sleep behaviours. Incident fall and dementia were identified through linkage to health records and the death registry up to December 2022. Multistate time-to-event analysis was conducted to examine the associations between activity profiles and the risks of fall, dementia, and their transitions over time.

**Results:**

Based on various activity levels and work status, participants were categorized into four groups: active-non-working (29.2%), inactive-non-working (14.4%), active-working (26.7%), and inactive-working (29.7%). Of them, 19,753 (6.85%) experienced a fall injury, and 4,407 (1.53%) developed dementia over an average follow-up of 13.4 years. The inactive-non-working group showed the highest risk of both fall and dementia (HR_range_: 1.45—2.38) over 13.4 years. In the non-working groups, where fall and dementia occurred most often, the inactive profile was linked to a higher risk of both fall and dementia, and to an increased likelihood of transitioning between the two. Transitions from dementia to subsequent fall were associated with insufficient physical activity, whereas transitions from fall to subsequent dementia were linked to social isolation, particularly living alone and frequent loneliness. In the working groups, no differences in fall or dementia risk were observed between active and inactive profiles.

**Conclusions:**

This study highlights the important role of social and physical activity in simultaneously preventing fall, dementia, and their transitions, particularly among non-working individuals, including those on sick leave or retired.

**Supplementary Information:**

The online version contains supplementary material available at 10.1186/s13195-026-02140-2.

## Background

Fall and dementia are significant public health concerns in aging populations, often sharing overlapping risk factors [1]. Approximately a quarter of older adults aged ≥ 65 experience a fall annually [2, 3], while individuals with dementia are 2—3 times more likely to experience fall compared to those cognitively normal, with an annual incidence of 60—80% [3, 4]. However, previous evidence has largely focused on the incidence of fall and dementia separately, with less attention given to their co-occurrence, specifically the transitions from dementia- or fall-only states to the combined occurrence of both conditions over time, such as from dementia to subsequent fall, or from fall to subsequent dementia. The link between fall and dementia may stem from the long preclinical phase that begins years or even decades before diagnosis [5]. For example, motoric cognitive risk syndrome, characterized by slow gait and subjective cognitive complaints [6, 7], predicts both dementia and increased fall risk [1, 6, 7]. In addition, fall may act as a sentinel event for future cognitive decline and dementia risk, an area that has been largely underexplored [1, 8, 9]. Therefore, identifying associated protective factors may become crucial for developing effective dual prevention strategies for fall and dementia.

An active lifestyle is associated with a lower risk of both fall and dementia. Greater engagement in physical activities, less sedentary behaviour, and healthy sleep behaviour have been observed to contribute to this protective effect [10–13]. Identifying optimal activity profiles, even in midlife, may aid in preventing both fall and dementia. However, research gaps remain in defining optimal activity profiles. Most previous research has focused on single domains and overlooked interactions between leisure and non-leisure activities [14]. Specifically, leisure-time social activity (SA), particularly when combined with physical activity, plays a fundamental role in healthy aging by reducing dementia and fall risks [15–17], for example through increased production of neurotrophic factors and improved psychological well-being [16, 18, 19]. Yet, SA is often ignored in defining activity profiles. Additionally, active commuting may contribute to overall activity levels and have a positive effect on cognition, although evidence in older adults is limited [20]. Occupational activity has been associated with cognitive outcomes, with evidence suggesting that intermediate or high occupational physical activity is linked to an increased risk of cognitive impairment [21]. Moreover, occupational and leisure-time physical activity tend to be inversely related, with individuals in physically demanding occupations being less likely to engage in physical activity during leisure time [22]. However, no study has examined multidimensional, data-driven activity profiles — integrating diverse activity and behavioural domains — and their association with transitions in combined fall-dementia status over time.

Using data from the UK Biobank study of 288,287 adults aged 40 years and older, this study aimed first to identify distinct activity profiles and then to investigate their associations with the risk of fall, dementia, and the dynamic transitions between these states over time.

## Methods

### Study design and participants

This prospective longitudinal study was based on the UK Biobank data, which was collected at baseline from over 500,000 participants aged ≥ 40 years between 2006 and 2010 [23]. Participants attended a baseline examination at one of 22 assessment centres across the UK, where they completed a structured interview and touch-screen questionnaires, provided biological samples, and underwent physical and functional assessments. The follow-up health information was obtained through linkages with national registries, including primary care records, hospital admissions, and death registries [23, 24].

From the total sample of 502,198 participants, we excluded those with a diagnosis of dementia (*n* = 257) or any recorded fall event (*n* = 9,538) prior to baseline. Participants without follow-up information (*n* = 36,829) or with missing data on any activity profile measure (*n* = 167,287) were also excluded, resulting in a final analytical sample of 288,287 participants (*Supplementary Fig. 1*). Detailed information is provided in the Supplementary Method.

### Measurements for activity profiles

*Physical activity* was measured using the short-form International Physical Activity Questionnaire (IPAQ), which captures a composite score of work-related, transport, domestic, and leisure-time physical activities [25]. A combined moderate-to-vigorous physical activity score (metabolic task equivalents [MET]-min/week) was calculated [26] to categorize participants according to the World Health Organization (WHO) activity guidelines: not meeting (< 600 MET-min/week), meeting (600—1200 MET-min/week), or exceeding (> 1200 MET-min/week) the recommendations [27].

*Sleep behaviours* were assessed using a composite score based on five sleep factors. Low-risk sleep behaviour was defined by the following characteristics: early chronotype (“morning” or “more morning than evening”), 7—8 h of sleep per day, no or rare insomnia, absence of snoring, and no frequent daytime sleepiness [28]. Participants received 1 point for each healthy factor and 0 points otherwise, yielding a total sleep score ranging from 0 to 5, with higher scores indicating healthier sleep patterns.

We used *social isolation* as a proxy for *SA*, encompassing both functional and structural aspects [29]. Functional isolation was defined as reporting either never being able to confide in someone close or experiencing frequent feelings of loneliness; no functional isolation was defined as the absence of both indicators. Structural social isolation was defined as having less than monthly contact with friends or family, no weekly participation in group activities, or living alone; no structural isolation was defined as the absence of all these indicators [29].

*Transport activity* was assessed based on commuting mode and occupational status. Participants were first divided into working and non-working groups. The working group was then further categorized into inactive commuters (using car or public transport) and active commuters (walking, cycling, or using a mixed mode of transport, such as walking combined with a bus or cycling combined with a train) [30]. Finally, transport activity was classified into three categories: non-working, inactive commuters, and active commuters.

*Occupational activity* was evaluated based on participants’ occupational backgrounds [31]. Manual labourers were defined as those reporting job-related physical demands occurring ‘sometimes’, ‘usually’, or ‘always’. Similar to transport activity, occupational activity was classified into three groups based on occupational status and job-related physical demands: non-working group, non-manual workers, and manual workers.

### Status of all-cause dementia, fall incidents, and mortality

All-cause dementia cases in the UK Biobank were identified from multiple sources during follow-up through December 2022. These resources included algorithmically defined health outcomes, first-occurrence data for dementia onsets, death registry, hospital inpatient records, and primary care data [32]. Diagnoses were classified according to International Classification of Diseases, 10th Revision (ICD-10) codes for all-cause dementia, specifically A81, F00, F01, F02, F03, F05, F10, G30, G31, and I67.

Fall status was defined by the occurrence of a first inpatient-recorded injurious fall. Accordingly, the fall state corresponds to the post-first injurious fall period, with individuals remaining in this state after their first recorded injurious fall. Fall events were identified through linkage to inpatient hospital admission data using ICD-10 codes W00, W01, W05—W10, and W17—W19. Consistent with previous aging-related research [33], falls resulting from external causes or high-energy trauma were excluded to focus on age-related falls. Death status was obtained via linkage to the death register, with dates and causes of death recorded and updated through 31 ^st^ December 2022.

### Statistical analysis

Baseline characteristics by fall, dementia, and their co-occurrence were reported as frequencies (n, %), means (SD) for normally, and medians (IQR) for non-normally continuous variables. Group differences were assessed with chi-square tests for categorical variables, ANOVA for normally distributed, and Kruskal-Wallis tests for non-normally distributed continuous variables.

To capture different activity profiles, we performed Latent Class Analysis (LCA), a person-centred approach to identify the minimum number of classes that describe underlying patterns in the data and determine each individual’s likelihood of belonging to each class [34, 35]. Six categorical variables (i.e., physical activity, sleep behaviour, functional isolation, structural isolation, transport activity, and occupational activity) were introduced into the model. Model performance was assessed according to the Bayesian Information Criterion (BIC), Akaike Information Criterion (AIC), their respective reductions, smallest class size, entropy, and average posterior probability of assignment. Baseline characteristics of identified activity profiles were further compared using chi-square, ANOVA, or Kruskal-Wallis tests.

**Fig. 1:**
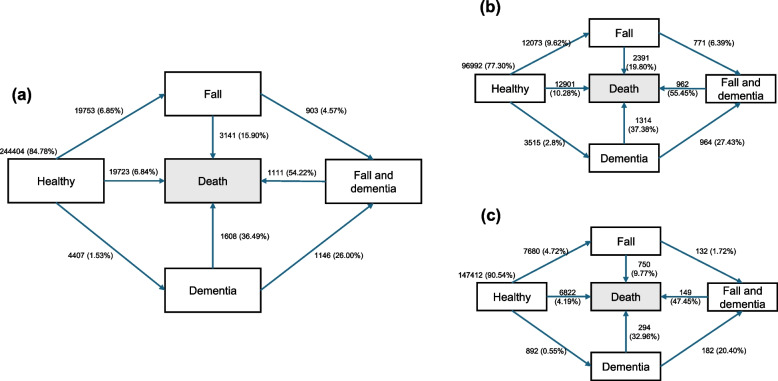
Transitions from healthy to fall, dementia, or death, shown as numbers and percentages of participants: **a** total sample, **b** non-working profiles, **c** working profiles. *Notes*. This figure displays the raw descriptive statistics for the study cohort, specifically displaying transition counts (the total number of individuals moving between states over the follow-up period) and crude proportions. For example, in the total sample (**a**), 84.78% of individuals remained healthy until the end of the follow-up period, while 6.85% experienced fall, 1.53% developed dementia, and 6.84% died. Among the 19,753 individuals who experienced fall, 4.57% subsequently developed dementia and 15.9% died. Of the 4,407 individuals who developed dementia, 26.0% later experienced fall and 36.49% died. Among those who experienced both fall and dementia, 54.22% died. Corresponding numbers and proportions are shown for non-working (**b**) and working (**c**) profiles

Multistate models were employed to estimate the dynamic progression between multiple health outcomes, with hazard ratios (HRs) and 95% confidence intervals (CIs) calculated for each transition [36]. Distinct, mutually exclusive states were specified as outcomes: healthy; dementia without fall (hereafter referred to as “dementia”), fall without dementia (hereafter “fall”, referring to the first fall recorded), dementia followed by fall or fall followed by dementia (hereafter “fall and dementia”), and death. The model included eight possible state transitions (Fig. 1a): (1) from healthy to fall, (2) from healthy to dementia, (3) from healthy to death, (4) from fall to fall and dementia, (5) from fall to death, (6) from dementia to fall and dementia, (7) from dementia to death, (8) from fall and dementia to death. We identified the temporal order of fall and dementia using the recorded diagnosis dates from the patient registers. Multistate models allow estimation of state-specific hazard rates while accounting for the time-varying nature of transitions and competing risks. Activity profiles identified by LCA were introduced as exposures to estimate their impact on the risk of progression between states. Follow-up time was calculated from the baseline visit date to the date of transition to the respective states or the date of the latest data availability (31 December 2022). If dementia or a fall occurred on the same date as death, 0.5 days were added to the death date. Similarly, if dementia and a fall were diagnosed on the same date, 0.5 days were added to the fall date, assuming that dementia pathology preceded the fall diagnosis [26, 37]. Models were adjusted for age, sex, education, Charlson Comorbidity Index (CCI), hearing, vision, handgrip strength, and heavy drinking, while transitions involving dementia were additionally adjusted for *APOE* ε4 status. The assessment of the covariates is described in the *Supplementary Method.* These covariates were chosen based on the assumptions about their relationships with exposure and outcomes, which are illustrated in a directed acyclic graph (DAG) in *Supplementary Fig. 2*. All analyses were first conducted in the full sample, followed by stratified analyses based on activity profiles.

In the sensitivity analyses, we excluded dementia and fall events occurring within 3 years and 10 years of baseline to address the potential impact of reverse causality. We additionally adjusted for other covariates and distal ancestors identified in the DAG in the primary analysis to evaluate the robustness of the primary results. Moreover, non-working individuals who were not retired were excluded, as retirees (81%) likely represent a distinct profile compared with those who were unemployed or on sick leave. We additionally performed a sensitivity analysis incorporating posterior probabilities of class membership to evaluate whether the main findings were robust to potential misclassification.

All statistical analyses were performed in R (version 4.2.2). LCA was conducted using the poLCA package, while the mstate and survival packages were used to fit the multistate models and estimate HRs.

## Results

### Baseline characteristics of the study population

The median age of study participants was 58.4 years (IQR: 50.5—63.8 years) and 53.6% were women. Over a mean follow-up of 13.4 years, participants were classified into four transition states: healthy (*n* = 264,127), dementia (*n* = 3,261), fall injuries (*n* = 18,850), and both dementia and fall injuries (*n* = 2,049). Table 1 presents the baseline characteristics of study participants across the four transition states. In brief, compared to healthy participants or those with only fall injuries, individuals with dementia or with both fall and dementia were more likely to be older, men, have lower education, carry the *APOE* ε4 allele, live alone or experience social isolation, not employed, and have more vascular risk factors — such as overweight or obesity, elevated systolic blood pressure, and current or past smoking — as well as vision or hearing impairments. In contrast, participants with only fall injuries tended to have lower grip strength than the other groups.

**Table 1:** Baseline characteristics of study participants by dynamic transition states

Characteristics	Total sample	Dynamic transition states				
		Healthy individuals	Incident dementia or fall			*p***-**value
			Dementia	Fall	Both	
	(*n* = 288287)	(*n* = 264127)	(*n* = 3261)	(*n* = 18850)	(*n* = 2049)	
Age (years), median (IQR)	58.4 (50.5—63.8)	57.8 (50.0—63.3)	65.7 (62.3—68.1)	62.0 (56.0—66.1)	66.1 (63.4—68.3)	< 0.001
Women, n (%)	154634 (53.6)	140309 (53.1)	1398 (42.9)	11969 (63.5)	958 (46.8)	< 0.001
Education, n (%)						
University or higher	98494 (34.2)	91928 (34.8)	736 (22.6)	5416 (28.7)	414 (20.2)	< 0.001
Below University	188209 (65.3)	170811 (64.7)	2486 (76.2)	13310 (70.6)	1602 (78.2)	
Missing	1584 (0.5)	1388 (0.5)	39 (1.2)	124 (0.7)	33 (1.6)	
***APOE***ε4 status, n (%)						
ε4 carriers	68057 (23.6)	61439 (23.3)	1467 (45.0)	4298 (22.8)	853 (41.6)	< 0.001
Non-ε4 carriers	170491 (59.1)	157363 (59.6)	1214 (37.2)	11089 (58.8)	825 (40.3)	
Missing	49739 (17.3)	45325 (17.2)	580 (17.8)	3463 (18.4)	371 (18.1)	
Physical activity categories, n (%)						
< 600 METs	113601 (39.4)	103469 (39.2)	1298 (39.8)	7938 (42.1)	896 (43.7)	< 0.001
600—1200 METs	50997 (17.7)	47043 (17.8)	523 (16.0)	3102 (16.5)	329 (16.1)	
> 1200 METs	123689 (42.9)	113615 (43.0)	1440 (44.2)	7810 (41.4)	824 (40.2)	
Sleep score, n (%)						
0	679 (0.2)	601 (0.2)	6 (0.2)	66 (0.4)	6 (0.3)	< 0.001
1	12132 (4.2)	10,913 (4.1)	173 (5.3)	952 (5.1)	94 (4.6)	
2	56700 (19.7)	51,606 (19.5)	638 (19.6)	4010 (21.3)	446 (21.8)	
3	110284 (38.3)	100886 (38.2)	1230 (37.7)	7372 (39.1)	796 (38.8)	
4	87728 (30.4)	80853 (30.6)	949 (29.1)	5339 (28.3)	587 (28.6)	
5	20764 (7.2)	19268 (7.3)	265 (8.1)	1111 (5.9)	120 (5.9)	
Functional Isolation, n (%)	75 469 (26.2)	68 048 (25.8)	1002 (30.7)	5713 (30.3)	706 (34.5)	< 0.001
Structural Isolation, n (%)	128173 (44.5)	116554 (44.1)	1555 (47.7)	9010 (47.8)	1054 (51.4)	< 0.001
Transport Activity, n (%)						
Not working	125481 (43.5)	109893 (41.6)	2551 (78.2)	11302 (60.0)	1735 (84.7)	< 0.001
Inactive	126974 (44.0)	120161 (45.5)	589 (18.1)	5970 (31.7)	254 (12.4)	
Active	35832 (12.4)	34073 (12.9)	121 (3.7)	1578 (8.4)	60 (2.9)	
Occupational Activity, n (%)						
Not working	125481 (43.5)	109893 (41.6)	2551 (78.2)	11302 (60.0)	1735 (84.7)	< 0.001
Non-manual	112760 (39.1)	106915 (40.5)	437 (13.4)	5204 (27.6)	204 (10.0)	
Manual	50046 (17.4)	47319 (17.9)	273 (8.4)	2344 (12.4)	110 (5.4)	
Smoking status, n (%)						
Current	29043 (10.1)	26188 (9.9)	298 (9.1)	2341 (12.4)	216 (10.5)	< 0.001
Former	102841 (35.7)	93447 (35.4)	1461 (44.8)	7035 (37.3)	898 (43.8)	
Never	155759 (54.0)	143927 (54.5)	1495 (45.8)	9415 (49.9)	922 (45.0)	
Missing	644 (0.2)	565 (0.2)	7 (0.2)	59 (0.3)	13 (0.6)	
BMI status, n (%)						
Underweight (BMI < 18.5)	1409 (0.5)	1227 (0.5)	18 (0.6)	144 (0.8)	20 (1.0)	< 0.001
Normal (18.5 ≤ BMI < 25)	94167 (32.7)	86 572 (32.8)	926 (28.4)	6052 (32.1)	617 (30.1)	
Overweight (25 ≤ BMI < 30)	123228 (42.7)	113431 (42.9)	1476 (45.3)	7477 (39.7)	844 (41.2)	
Obese (BMI ≥ 30)	68188 (23.7)	61787 (23.4)	815 (25.0)	5039 (26.7)	547 (26.7)	
Missing	1295 (0.4)	1110 (0.4)	26 (0.8)	138 (0.7)	21 (1.0)	
SBP (mmHg), mean (SD)	137.6 (18.6)	137.3 (18.5)	144.0 (19.5)	139.7 (19.3)	144.2 (19.5)	< 0.001
Grip strength (kilograms), mean (SD)	61.9 (21.9)	62.5 (21.9)	58.3 (21.4)	54.7 (20.3)	55.3 (20.4)	< 0.001
Vision problem, n (%)						
Yes	44642 (15.5)	39785 (15.1)	748 (22.9)	3646 (19.3)	463 (22.6)	< 0.001
No	242483 (84.1)	223321 (84.6)	2487 (76.3)	15108 (80.1)	1567 (76.5)	
Missing	1162 (0.4)	1021 (0.4)	26 (0.8)	96 (0.5)	19 (0.9)	
Hearing problem/disorder, n (%)						
Yes	64106 (22.2)	58216 (22.0)	911 (27.9)	4425 (23.5)	554 (27.0)	< 0.001
Yes, with hearing aid	8630 (3.0)	7433 (2.8)	229 (7.0)	818 (4.3)	150 (7.3)	
No	206195 (71.5)	189887 (71.9)	2021 (62.0)	13008 (69.0)	1279 (62.4)	
Missing	9356 (3.2)	8591 (3.3)	100 (3.1)	599 (3.2)	66 (3.2)	

Physical activity categories were defined based on weekly moderate-to-vigorous physical activity, expressed in metabolic equivalent of task (MET) units, and classified according to the World Health Organization’s weekly physical activity recommendations

*Abbreviations: IQR* Interquartile Range, *SD* Standard Deviation, *BMI* body mass index, *MET* Metabolic Equivalent of Task

### Activity profiles identified by LCA

**Fig. 2:**
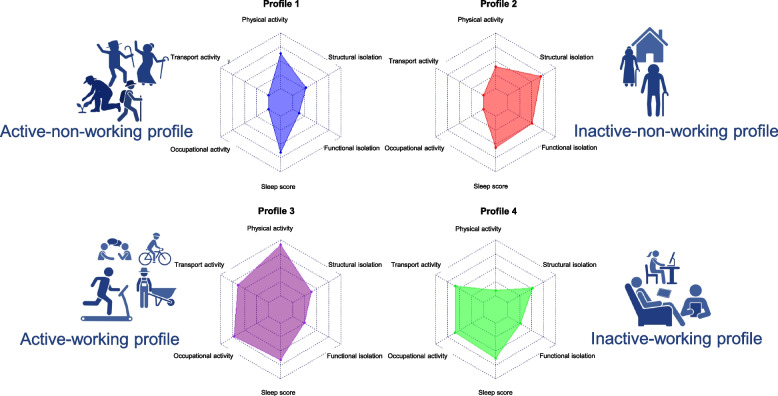
Radar plot of mean activity and behavioural factors by the four latent profiles. *Notes*. Illustrations represent the association between individual activity factors and the four activity profiles. This radar plot serves as a tool for comparing multidimensional activity profiles and does not inherently display statistical significance. Categorical or ordinal variables, including physical activity, transport activity, occupational activity, sleep score, functional isolation, and structural isolation, are demonstrated here as individual activity factors.The radar plot displays the mean values of activity and behavioural factors for each latent profile/class. Greater distance from the center indicates higher levels of physical activity, healthier sleep scores, greater likelihood of functional and structural social isolation, active commuting, and engagement in manual work

Based on the predetermined selection criteria (*Supplementary Table 1*), we identified a four-class solution by integrating all individual activity factors. All participants were classified into four groups based on their working status and activity levels, defined as: (1) active-non-working profile (29.2%), (2) inactive-non-working profile (14.4%), (3) active-working profile (26.7%), and (4) inactive-working profile (29.7%). Each activity profile and its association with individual activity factors are illustrated in Fig. 2. Baseline characteristics between different activity profiles are presented in *Supplementary Table 2*. Compared to the working profiles (profiles 3 and 4), individuals with non-working profiles (profiles 1 and 2) were more likely to be older, have lower educational attainment, higher systolic blood pressure, and reduced grip strength. Compared to the other three profiles, the inactive-non-working profile (profile 2) had the highest proportion of smokers, overweight and obese individuals, and the greatest prevalence of vision and hearing problems, but a lower proportion of *APOE* ε4 carriers. The active-working profile included a higher proportion of men than the other groups.

### Dynamic transitions between dementia and fall in relation to activity profiles

Over an average follow-up of 13.4 years, among 288,287 participants, 19,753 (6.85%) experienced a fall injury, 4,407 (1.53%) developed dementia, and 19,723 (6.84%) died without either event (Fig. 1a). In addition, 4.57% of individuals with a fall injury subsequently developed dementia, 26.0% of those with dementia later experienced a fall, and more than half of those with both events died. Notably, the average time from a healthy state to the first event — either a fall (7.88 ± 3.92 years) or dementia (9.70 ± 3.12 years) — was longer than the subsequent transitions to co-occurrence from fall to subsequent dementia (3.41 ± 3.08 years) or from dementia to subsequent fall (0.96 ± 1.65 years).

A higher proportion of individuals with non-working profiles experienced a fall injury (9.62% vs. 4.72%) and developed dementia (2.8% vs. 0.55%) compared with those with working profiles (Fig. 1b vs. c). Furthermore, those with non-working profiles had a higher proportion of transitions to co-occurrence, including from fall to subsequent dementia (6.39% vs. 1.72%), from dementia to subsequent fall (27.43% vs. 20.40%), and a higher mortality after experiencing both events (55.45% vs. 47.45%) (Fig. 1b vs. c).

We performed the multi-state model analyses in full-sample and subgroup models. In the full-sample model, the active-working profile served as the reference group, and the other three groups were compared against it. This allowed us to examine whether the active-working and active-non-working profiles differ, whether the active-working and inactive-working profiles differ, and whether there is evidence of an ordered pattern across the four groups. Given that most transitions occurred within the non-working group, we subsequently restricted the analysis to this subgroup. In this subgroup model, we compared the inactive and active profiles among non-working individuals to assess whether they are associated with different transition patterns. In the full-sample model, our results showed that, compared with individuals in the active-working profile, those in the inactive-working profile did not differ significantly across any transitions (Table 2). In contrast, individuals in the non-working profiles had a higher risk of fall, dementia, and mortality, as well as higher risks of transitioning from fall to subsequent dementia and from fall to death, with the inactive- non-working profile showing the highest risk across outcomes (Table 2). In the subgroup model, compared with the active-non-working profile, individuals with inactive-non-working profile had an increased risk of fall (HR: 1.31; 95% CI: 1.26—1.36), dementia (HR: 1.22; 95% CI: 1.14—1.31), transitions from fall to subsequent dementia (HR: 1.27; 95% CI: 1.10—1.47), and transitions from dementia to subsequent fall (HR: 1.19; 95% CI: 1.04—1.37) (Table 2). In addition, compared with the active-non-working group, the inactive-non-working group had a higher risk of mortality both from death without experiencing either fall or dementia (HR: 1.39; 95% CI: 1.34—1.44) and from transitions from fall to death (HR: 1.30; 95% CI: 1.20—1.41).

**Table 2:** Hazard ratios (HRs) and 95% confidence intervals (CIs) for multistate transitions across health, fall, dementia, and death by activity profile in the full sample and in non-working groups

Full sample (*n* = 288,287)								
	Healthy to fall (*n* = 19 753)^a^	Healthy to dementia (*n* = 4407)^b^	Healthy to death (*n* = 19 723)^a^	Fall to fall & dementia (*n* = 903)^b^	Fall to death (*n* = 3141)^a^	Dementia to fall & dementia (*n* = 1146)^b^	Dementia to death (*n* = 1608)^b^	Fall & dementia to death (*n* = 1111)^b^
	HRs (95% CI)	HRs (95% CI)	HRs (95% CI)	HRs (95% CI)	HRs (95% CI)	HRs (95% CI)	HRs (95% CI)	HRs (95% CI)
Active-working	Ref	Ref	Ref	Ref	Ref	Ref	Ref	Ref
Inactive-working	1.01 (0.96 to 1.05)	1.01 (0.89 to 1.16)	1.03 (0.98 to 1.08)	1.37 (0.96 to 1.94)	1.06 (0.91 to 1.22)	0.94 (0.70 to 1.25)	1.06 (0.85 to 1.34)	1.13 (0.82 to 1.57)
Active-non-working	1.10 (1.05 to 1.15)^***^	1.39 (1.24 to 1.55)^***^	1.15 (1.10 to 1.20)^***^	1.86 (1.38 to 2.52)^***^	1.26 (1.11 to 1.43)^***^	1.02 (0.81 to 1.29)	1.07 (0.88 to 1.29)	1.08 (0.83 to 1.42)
Inactive-non-working	1.45 (1.38 to 1.52)^***^	1.69 (1.51 to 1.91)^***^	1.62 (1.54 to 1.70)^***^	2.38 (1.75 to 3.22)^***^	1.64 (1.44 to 1.87)^***^	1.21 (0.95 to 1.54)	1.02 (0.83 to 1.25)	1.07 (0.81 to 1.40)

Non-working profiles (***n*** = 125481)								
	Healthy to fall (*n* = 12 073)^a^	Healthy to dementia (*n* = 3515)^b^	Healthy to death (*n* = 12 901)^a^	Fall to fall & dementia (*n* = 771)^b^	Fall to death (*n* = 2391)^a^	Dementia to fall & dementia (*n* = 964)^b^	Dementia to death (*n* = 1314)^b^	Fall & dementia to death (*n* = 962)^b^
	HRs (95% CI)	HRs (95% CI)	HRs (95% CI)	HRs (95% CI)	HRs (95% CI)	HRs (95% CI)	HRs (95% CI)	HRs (95% CI)
Active profile	Ref	Ref	Ref	Ref	Ref	Ref	Ref	Ref
Inactive profile	1.31 (1.26 to 1.36)^***^	1.22 (1.14 to 1.31)^***^	1.39 (1.34 to 1.44)^***^	1.27 (1.10 to 1.47)^**^	1.30 (1.20 to 1.41)^***^	1.19 (1.04 to 1.37)^*^	0.96 (0.85 to 1.08)	0.99 (0.87 to 1.13)

*HR* hazard ratio, *CI* confidence interval

^a^Model was adjusted for age, sex, education, Charlson Comorbidity Index, hearing, vision, handgrip strength and heavy drinking

^b^Model was adjusted for age, sex, education, Charlson Comorbidity Index, hearing, vision, handgrip strength, heavy drinking, and presence of *APOE ε*4 allele

^*^P < 0.05; ^**^P < 0.01; ^***^P < 0.001

Among the non-working groups, we further examined the contributions of individual activity factors and their defining components to incident fall, dementia, and their transitions to co-occurrence. As transport-related and occupational activities were not applicable in this group, analyses focused on four individual activity factors: physical activity (moderate and vigorous levels), structural social isolation (infrequent contact with friends or family, absence of weekly group activities, and living alone), functional social isolation (never able to confide in others, and frequent loneliness), and sleep score (early chronotype, sleep duration, insomnia, snoring, and daytime sleepiness). Table 3 displays that among the four individual activity factors, a low level of moderate-to-vigorous physical activity (< 600 MET-min/week), presence of functional and structural social isolation, and poorer sleep behaviours were all associated with incident fall, dementia, and mortality. Low physical activity was additionally associated with transitions from dementia to subsequent fall, whereas social isolation was more strongly associated with transitions from fall to subsequent dementia. Consistent with the results for the four activity factors, all their defining components were associated with incident fall, dementia, and mortality (*Supplementary Table S3*). Notably, living alone, frequent feelings of loneliness, and not sleeping 7–8 h per day were significantly associated with increased risk of transitions from fall to subsequent dementia. In addition to insufficient physical activity components, insomnia was also associated with an increased risk of transitions from dementia to subsequent fall.

**Table 3:** Hazard ratios (HRs) and 95% confidence intervals (CIs) for multistate transitions across health, fall, dementia, and death according to physical activity, social isolation, and sleep score among non-working individuals

Non-working profiles (*n* = 125481)								
	**Healthy to fall (*n* = 12 073)**^**a**^	**Healthy to dementia (*n* = 3515)**^**b**^	**Healthy to death (*n* = 12 901)**^**a**^	**Fall to fall & dementia (*n* = 771)**^**b**^	**Fall to death (*n* = 2391)**^**a**^	**Dementia to fall & dementia (*n* = 964)**^**b**^	**Dementia to death (*n* = 1314)**^**b**^	**Fall & dementia to death (*n* = 962)**^**b**^
	HRs (95% CI)	HRs (95% CI)	HRs (95% CI)	HRs (95% CI)	HRs (95% CI)	HRs (95% CI)	HRs (95% CI)	HRs (95% CI)
Total physical activity categories								
600—1200 MET-min/week	Ref	Ref	Ref	Ref	Ref	Ref	Ref	Ref
> 1200 MET-min/week	1.00 (0.95, 1.05)	0.99 (0.90, 1.09)	0.95 (0.90, 1.00)^*^	0.91 (0.74, 1.12)	1.08 (0.95, 1.22)	0.99 (0.83, 1.20)	0.92 (0.78, 1.07)	0.79 (0.66, 0.95)^*^
< 600 MET-min/week	1.18 (1.12, 1.24)^***^	1.12 (1.02, 1.23)^*^	1.26 (1.20, 1.32)^***^	1.09 (0.89, 1.33)	1.33 (1.18, 1.51)^***^	1.21 (1.00, 1.46)^*^	1.03 (0.88, 1.21)	0.86 (0.72, 1.04)
Functional social isolation								
Not present	Ref	Ref	Ref	Ref	Ref	Ref	Ref	Ref
Present	1.23 (1.18, 1.28)^***^	1.28 (1.19, 1.37)^***^	1.20 (1.16, 1.25)^***^	1.28 (1.11, 1.49)^***^	1.10 (1.01, 1.19)^*^	1.07 (0.94, 1.23)	0.88 (0.78, 0.99)^*^	1.06 (0.93, 1.21)
Structural social isolation								
Not present	Ref	Ref	Ref	Ref	Ref	Ref	Ref	Ref
Present	1.22 (1.18, 1.27)^***^	1.19 (1.11, 1.27)^***^	1.30 (1.26, 1.35)^***^	1.31 (1.14, 1.52)^***^	1.29 (1.19, 1.40)^***^	1.08 (0.95, 1.23)	1.02 (0.91, 1.14)	0.82 (0.72, 0.93)^**^
Sleep score	0.93 (0.92, 0.95)^***^	1.02 (0.99, 1.06)	0.93 (0.92, 0.95)^***^	0.97 (0.90, 1.04)	0.94 (0.90, 0.98)^**^	1.01 (0.94, 1.07)	1.05 (0.99, 1.11)	1.07 (1.00, 1.14)^*^

Physical activity categories were defined based on weekly moderate-to-vigorous physical activity, expressed in metabolic equivalent of task (MET) units, and classified according to the World Health Organization’s weekly physical activity recommendations

*HR* hazard ratio, *CI* confidence interval, *MET* metabolic task equivalent

^a^Model was adjusted for age, sex, education, multimorbidity, hearing, vision, handgrip strength and heavy drinking

^b^Model was adjusted for age, sex, education, multimorbidity, hearing, vision, handgrip strength, heavy drinking, and presence of APOE4-allele

^*^P < 0.05; ^**^P < 0.01; ^***^P < 0.001

### Sensitivity analyses

The findings remained consistent after excluding individuals who developed fall or dementia within three years of baseline, and were similarly robust when applying a 10-year exclusion period (*Supplementary Tables 4–7*). The primary results remained the same when we additionally adjust for other covariates identified in DAGs (*Supplementary Tables 8–9*).

After excluding non-retired individuals from the non-working group, the active-non-working profile in the full-sample model no longer showed increased risks of falling, mortality, or post-fall mortality compared with the active-working profile. This finding suggests that other forms of work absence, such as sick leave, may have contributed to these associations (*Supplementary Table 10*). The inactive-non-working profile retained all previously observed associations compared with the active working profile and additionally showed a significantly increased risk of transitioning from dementia to fall and dementia (*Supplementary Table 10*). When the analysis was restricted to retirees in the non-working subgroup, the findings remained consistent, with low physical activity, social isolation, and unhealthy sleep behaviours emerging as key factors associated with both fall and dementia in this group (*Supplementary Table 11*). When posterior probabilities of class membership were incorporated into the main analysis, results in the full-sample model showed that, compared with the active-working profile, the active-non-working profile did not differ significantly across any transitions. The inactive-working profile was associated with an increased risk of dementia and a higher likelihood of transitioning from a fall to subsequent dementia. The inactive-non-working profile consistently showed the highest risks across most outcomes, including fall, dementia, mortality, fall-to-death transitions, and transitions between fall and dementia states (*Supplementary Table 12*).

## Discussion

The key findings of this study can be summarized as follows. First, we identified four distinct activity profiles among adults aged 40 to 69 years: (1) active-non-working, (2) inactive-non-working, (3) active-working, and (4) inactive-working. Second, incident fall and dementia, along with their transitions, occurred more frequently in non-working profiles (both active and inactive) than in working profiles. The inactive-non-working group exhibited the highest risks for fall, dementia, and their transitions. Third, lack of physical activity was associated with increased likelihood of transitions from dementia to subsequent fall, while social isolation, particularly living alone and feelings of loneliness, was more strongly associated with transitions from fall to subsequent dementia.

Recent research increasingly employs data-driven approaches to identify behavioural subgroups, shifting from single lifestyle factors to a holistic view that acknowledges the combined influence of multiple behaviours on dementia and fall risk. Consistent with our findings, previous studies clustering similar lifestyle behaviours in the context of cognitive ageing often identify profiles characterized by uniformly low or high engagement across all healthy behaviours [14, 17, 38]. This aligns with evidence showing that low participation in one healthy behaviour often correlates with low engagement in others [17]. Our study extends this understanding by differentiating activity profiles based on working status, identifying both active and inactive profiles in working and non-working populations. When we restricted the non-working group to retirees only, differences between the active-working profile and the inactive-non-working profile persisted, whereas the differences between the active-non-working and active-working profiles were largely attenuated and were no longer statistically significant for fall and death. When posterior probabilities of group membership were taken into account, differences between the active-non-working and active-working profiles were no longer observed for any of the outcomes. In our full sample, the active-non-working group likely reflects a heterogeneous mix of retirees and non-retired individuals out of work due to health or other reasons. Restricting the sample to retirees reduces this heterogeneity, suggesting that compositional differences may have contributed to the apparent excess risk in the active-non-working profile. This heterogeneity was further supported when posterior probabilities were incorporated, which may indicate uncertainty in profile membership and potential misclassification in class assignments. All these findings suggest that our main findings, particularly those involving the non-working profiles, should be interpreted with caution, as potential unmeasured confounding, misclassification, and selection effects (e.g., baseline health status, functional status, and socioeconomic position) cannot be fully ruled out. In addition, individuals with healthier lifestyles and better health are more likely to remain in the workforce. This is also consistent with previous studies showing that individuals outside the labour market tend to have poorer health, a higher burden of chronic diseases, and lower engagement in health-related behaviours [39, 40]. We further found that low physical activity, social isolation, and unhealthy sleep behaviour were key profile characteristics associated with both fall and dementia among non-working groups. When our analysis was restricted to retirees, these findings remained consistent.

Our study shows that while falls are more frequent than dementia incidence, individuals diagnosed with dementia were more likely to experience a fall afterward than those who fell were to develop dementia. These results may potentially be affected by a tie-breaking rule, particularly when dementia and a fall were recorded on the same date, as this could alter the inferred temporal ordering of events. In total, 534 participants had same-day diagnoses, for which such a rule was applied. Apart from this potential limitation, our results align with previous research using multistate models, which found that individuals were more likely to progress from cognitive impairment to cognitive frailty (a combination of cognitive impairment and physical frailty) than from physical frailty to cognitive frailty [41]. One study reported a 21% increased risk for dementia incidence following a fall injury [8], while another linked multiple falls to motoric cognitive risk syndrome but not to dementia incidence [1]. These discrepancies may stem from different measurements of fall, as self-reported falls often yield higher rates than injurious falls identified through hospital records. We identified fall injuries using ICD-10 codes related to external causes rather than isolated acute events [33]. The fall injuries were therefore considered as an indicator of geriatric syndrome and strongly associated with frailty and sarcopenia [42]. Analyses of transitions between fall injuries and dementia provide insight into the multiple pathways underlying geriatric syndromes. Our findings first demonstrated that 26% of individuals with dementia experienced an injurious fall after diagnosis, whereas fewer than 5% of those with an injurious fall later developed dementia. Furthermore, we found that low levels of physical activity and insomnia were associated with an increased risk of transitions from dementia to subsequent falls. This suggests that these behaviours may reinforce a brain-to-body vulnerability pathway, reducing physical resilience and making individuals with dementia more susceptible to physical decline and subsequent injury [43]. One possible explanation is that physical inactivity and insomnia may accelerate sarcopenia and influence neuromotor control. When cognitive impairment occurs, limited physical reserve may be insufficient to support functional capacity, increasing the risk of fall [43]. Insufficient sleep duration and social isolation, particularly loneliness and living alone, were linked to more transitions from fall to subsequent dementia. These findings underscore the bio-psycho-social nature of aging, where a physical event, such as a fall injury, can trigger subsequent neurological consequences and accelerate cognitive decline [8]. Greater SA reflects increased cognitive stimulation and cognitive reserve, which may help compensate for the impact of fall injuries on brain function. Insufficient sleep duration may impair glymphatic clearance and exacerbate neuroinflammation, thereby accelerating the transition from fall injuries to dementia.

To the best of our knowledge, this is the first study to investigate how data-driven activity profiles influence transitions to and between fall and dementia. Previous studies have identified lifestyle-based subgroups associated with cognitive outcomes, but none have explored the connections between fall and dementia. One study of 715 older adults identified three latent profiles — high, middle, and low participation in healthy lifestyle behaviours (physical activity, cognitive activity, healthy diet, SA) — and found that the high participation group had slower global cognitive decline [17]. A study of 2,059 older adults used six lifestyle domains (physical activity, SA, cognitive activity, sleep quality, life space, and social network) to derive five latent classes, showing that balanced engagement across domains was linked to slower cognitive decline and lower dementia risk [14]. Our findings highlight the potential role of activity patterns in preventing dual motor-cognitive decline. The consistent associations of both physical and social activities with reduced risks of dementia and fall underscore the importance of comprehensive, multidomain approaches to healthy ageing.

The strengths of this study include the large sample size and the long follow-up of the UK Biobank dataset. Additionally, LCA is a powerful method that allowed us to categorize distinct activity profiles within our study population [44]. This study examined transitions between incident fall (first occurrence) and dementia and assessed the impact of activity profiles using multistate models, accounting for the competing risk of all-cause mortality. However, several limitations exist. First, due to the prospective design of the cohort, activity profiles could only be assessed at baseline and not after each transition, limiting the ability to capture behavioural changes following a fall or dementia diagnosis. Second, measurements for the activity profiles were self-reported and might be subject to recall biases, e.g., people may overestimate their activity levels. Future studies employing objective activity measures are needed to verify these profiles. The short-form IPAQ questionnaires used in this study assessed physical activity across work, transportation, domestic chores, and leisure time. Although for the non-working group this primarily reflects leisure-time activity, it may still underestimate true leisure-time physical activity. Third, potential information bias, measurement error, and misclassification may have affected our findings. For example, individuals with cognitive impairment may inaccurately report self-reported activities and health-related behaviours, including sleep characteristics. This may partly explain the observed protective associations of some unhealthy behaviours with a lower risk of transitioning from dementia, or from fall and dementia to death. In addition, the non-working group was heterogeneous and likely included individuals with diverse reasons for non-employment (e.g., sick leave or health-related conditions), which may have contributed to uncertainty in profile assignment. As profile membership was assigned probabilistically, some degree of misclassification may have occurred. Fourth, the preclinical phase of dementia may occur decades before clinical diagnosis. During this period, changes in behaviours and psychosocial factors may emerge as a result of underlying cognitive decline, raising the possibility of reverse causality. We addressed this by excluding incident fall and dementia cases occurring within 3 and 10 years after baseline, and the results remained consistent. Fifth, fall injuries were identified through inpatient hospital admissions, meaning only injurious falls severe enough to require hospitalization were recorded, likely leading to an underestimation of overall fall incidence. Sixth, despite adjusting for confounders identified through the DAG and including additional covariates in sensitivity analyses, residual confounding from unmeasured factors in the complex relationship between fall, dementia, and activity profiles cannot be ruled out. Finally, UK Biobank participants tend to be healthier and less socioeconomically deprived than the general UK population, indicating a “healthy volunteer” selection bias that may limit the generalizability of our findings [45]. In addition, the analytic sample was further reduced due to missing behavioural data, such as physical activity. This represents an additional source of selection that may exacerbate healthy volunteer bias and further limit generalisability.

## Conclusions

Based on the UK Biobank data, we observed that nearly one in four individuals with dementia experienced a fall injury thereafter, and approximately 5% of individuals who experienced a fall injury subsequently developed dementia. These events occurred more frequently among non-working people, including those who were retired or on sick leave, and an inactive profile — characterized by low physical activity, high social isolation, and unhealthy sleep behaviour — was associated with an increased risk of incident dementia, fall, and their transitions. Our findings suggest that individuals with dementia may warrant particular attention to fall-risk assessment in clinical settings, given the observed transition pattern from dementia to subsequent fall injuries. Personalized intervention strategies may be particularly relevant for individuals who are not working, living alone, or experiencing loneliness, given the observed associations with transitions from fall injury to subsequent dementia. In addition, our findings support consideration of multidomain lifestyle interventions and programmes that address shared risk factors for both fall injuries and dementia.

## Supplementary Information

Supplementary Material 1.

## Data Availability

The data used in this study are available from the UK Biobank but cannot be made publicly available due to licensing restrictions. Qualified researchers may apply for access at: https://www.ukbiobank.ac.uk/enable-your-research/apply-for-access.

## Abbreviations

AD: Alzheimer’s disease
AIC: Akaike Information Criterion
*APOE* ε4: Apolipoprotein E ε4 allele
BIC: Bayesian Information Criterion
BMI: Body Mass Index
CCI: Charlson Comorbidity Index
CI: Confidence Interval
DAG: Directed Acyclic Graph
FAD: Familial Alzheimer’s disease
HR: Hazard Ratio
ICD-10: International Classification of Diseases, 10th Revision
IPAQ: International Physical Activity Questionnaire
IQR: Interquartile Range
LCA: Latent Class Analysis
MET: Metabolic Equivalent of Task
SA: Social Activity
SD: Standard Deviation
UK Biobank: United Kingdom Biobank
WHO: World Health Organization

## References

[1] Jayakody O, Blumen HM, Breslin M, Ayers E, Lipton RB, Verghese J, et al. Longitudinal associations between falls and future risk of cognitive decline, the Motoric Cognitive Risk syndrome and dementia: the Einstein Ageing Study. Age Ageing. 2022;51(3):afac058. 35290430 10.1093/ageing/afac058 PMC8923158

[2] Namoos, Thomson N, Bradley RS, Rudderman MAW, Aboutanos MMH. Memory Loss and Missteps: Investigating Fall Risks in Alzheimer's and Dementia Patients. 2024. 10.20900/agmr20240005 PMC11485985 39421020

[3] Simpkins C, Khalili SM, Yang F. Meta-analysis-based comparison of annual fall risk between older adults with Alzheimer’s disease and mild cognitive impairment. Adv Geriatr Med Res. 2024;6(1):e240002. 38725433 10.20900/agmr20240002 PMC11081206

[4] Härlein J, Dassen T, Halfens RJG, Heinze C. Fall risk factors in older people with dementia or cognitive impairment: a systematic review. J Adv Nurs. 2009;65(5):922–33. 19291191 10.1111/j.1365-2648.2008.04950.x

[5] Sperling RA, Aisen PS, Beckett LA, Bennett DA, Craft S, Fagan AM, et al. Toward defining the preclinical stages of Alzheimer’s disease: recommendations from the National Institute on Aging-Alzheimer’s Association workgroups on diagnostic guidelines for Alzheimer’s disease. Alzheimer’s Dementia. 2011;7(3):280–92. 21514248 10.1016/j.jalz.2011.03.003 PMC3220946

[6] Liang H, Fang Y. Longitudinal association between falls and motoric cognitive risk syndrome among community-dwelling older adults. Geriatric nursing (New York, NY). 2023;49:1–7. 10.1016/j.gerinurse.2022.11.003 36399977

[7] Beauchet O, Sekhon H, Schott A-M, Rolland Y, Muir-Hunter S, Markle-Reid M, et al. Motoric cognitive risk syndrome and risk for falls, their recurrence, and postfall fractures: results from a prospective observational population-based cohort study. J Am Med Dir Assoc. 2019;20(10):1268–73. 31201100 10.1016/j.jamda.2019.04.021

[8] Ordoobadi AJ, Dhanani H, Tulebaev SR, Salim A, Cooper Z, Jarman MP. Risk of dementia diagnosis after injurious falls in older adults. JAMA Netw Open. 2024;7(9):e2436606. 39348117 10.1001/jamanetworkopen.2024.36606 PMC11443352

[9] Wang Z, Wang Q, Fu C, Li X, Zhang L, Zhang X, et al. The fall-dementia connection: Synergistic effects of falls with genetic and health risk factors. J Alzheimers Dis. 2025;105(3):990–9. 40325974 10.1177/13872877251333799

[10] Bloomberg M, Brocklebank L, Hamer M, Steptoe A. Joint associations of physical activity and sleep duration with cognitive ageing: longitudinal analysis of an English cohort study. The Lancet Healthy Longevity. 2023;4(7):e345–53. 37421962 10.1016/S2666-7568(23)00083-1 PMC11883718

[11] Roe LS, Strotmeyer ES, Cawthon PM, Glynn NW, Ma Y, Ancoli-Israel S, et al. 24-hour activity composition is associated with lower fall and fracture risk in older men. J Bone Miner Res. 2024;40(1):27–37. 39348414 10.1093/jbmr/zjae160 PMC11700612

[12] Huang S-Y, Li Y-Z, Zhang Y-R, Huang Y-Y, Wu B-S, Zhang W, et al. Sleep, physical activity, sedentary behavior, and risk of incident dementia: a prospective cohort study of 431,924 UK Biobank participants. Mol Psychiatry. 2022;27(10):4343–54. 35701596 10.1038/s41380-022-01655-y

[13] Thibaud M, Bloch F, Tournoux-Facon C, Brèque C, Rigaud AS, Dugué B, et al. Impact of physical activity and sedentary behaviour on fall risks in older people: a systematic review and meta-analysis of observational studies. Eur Rev Aging Phys Act. 2012;9(1):5–15.

[14] Paolillo EW, Saloner R, VandeBunte A, Lee S, Bennett DA, Casaletto KB. Multimodal lifestyle engagement patterns support cognitive stability beyond neuropathological burden. Alzheimers Res Ther. 2023;15(1):221. 38111051 10.1186/s13195-023-01365-9 PMC10726589

[15] Dhakal U, McLaughlin S, Kim S, Roberts A, Vivoda J, Brown JS. Greater physical activity and social engagement offer protection against cognitive decline over time. Innov Aging. 2023;7(Supplement_1):1050.

[16] Jaqua EE, Tran M-LN, Alvarez P, Gupta M, Yoong J. Dementia and cognitive decline: a HEALM approach. Am J Lifestyle Med. 2024;19:779. 39540186 10.1177/15598276241291508 PMC11556629

[17] Halloway S, Wagner M, Tangney CC, Lange-Maia BS, Bennett DAA, Arvanitakis Z, et al. Latent Profile Analysis of Healthy Lifestyle Behaviors and its’ Association with Cognitive Decline in Older Adults. Alzheimers Dement. 2023;19(S22):e071672.

[18] Petersen N, König H-H, Hajek A. The link between falls, social isolation and loneliness: A systematic review. Arch Gerontol Geriatr. 2020;88:104020. 32018091 10.1016/j.archger.2020.104020

[19] Quach LT, Burr JA. Perceived social isolation, social disconnectedness and falls: the mediating role of depression. Aging Ment Health. 2021;25(6):1029–34. 32131617 10.1080/13607863.2020.1732294 PMC7483756

[20] Herfet M, Timperio A, Mazzoli E, Tittlbach S. Active life–active mind? Associations between active travel and cognitive functions across the lifespan: a systematic review. Cogent Soc Sci. 2024;10(1):2359632.

[21] Zotcheva E, Bratsberg B, Strand BH, Jugessur A, Engdahl BL, Bowen C, et al. Trajectories of occupational physical activity and risk of later-life mild cognitive impairment and dementia: the HUNT4 70+ study. Lancet Reg Health Eur. 2023;34:100721. 37927437 10.1016/j.lanepe.2023.100721 PMC10625024

[22] Iso-Markku P, Kujala UM, Knittle K, Polet J, Vuoksimaa E, Waller K. Physical activity as a protective factor for dementia and Alzheimer’s disease: systematic review, meta-analysis and quality assessment of cohort and case-control studies. Br J Sports Med. 2022;56(12):701–9. 35301183 10.1136/bjsports-2021-104981 PMC9163715

[23] Sudlow CLM, Gallacher J, Allen NE, Beral V, Burton P, Danesh J, et al. UK biobank: an open access resource for identifying the causes of a wide range of complex diseases of middle and old age. PLoS Med. 2015;12(3):e1001779. 25826379 10.1371/journal.pmed.1001779 PMC4380465

[24] Wilkinson T, Schnier C, Bush K, Rannikmäe K, Henshall DE, Lerpiniere C, et al. Identifying dementia outcomes in UK Biobank: a validation study of primary care, hospital admissions and mortality data. Eur J Epidemiol. 2019;34(6):557–65. 30806901 10.1007/s10654-019-00499-1 PMC6497624

[25] Craig CL, Marshall AL, Sjöström M, Bauman AE, Booth ML, Ainsworth BE, et al. International physical activity questionnaire: 12-country reliability and validity. Med Sci Sports Exerc. 2003;35(8):1381–95. 12900694 10.1249/01.MSS.0000078924.61453.FB

[26] Liu B-P, Zhu J-H, Wan L-P, Zhao Z-Y, Wang X, Jia C-X. The Impact of Physical Activity Intensity on the Dynamic Progression of Cardiometabolic Multimorbidity: Prospective Cohort Study Using UK Biobank Data. JMIR Public Health Surveill. 2023;9:e46991. 37747776 10.2196/46991 PMC10562971

[27] WHO guidelines on physical activity and sedentary behaviour: World Health Organization; 2020.

[28] Fan M, Sun D, Zhou T, Heianza Y, Lv J, Li L, et al. Sleep patterns, genetic susceptibility, and incident cardiovascular disease: a prospective study of 385 292 UK biobank participants. Eur Heart J. 2020;41(11):1182–9. 31848595 10.1093/eurheartj/ehz849 PMC7071844

[29] Foster HME, Gill JMR, Mair FS, Celis-Morales CA, Jani BD, Nicholl BI, et al. Social connection and mortality in UK Biobank: a prospective cohort analysis. BMC Med. 2023;21(1):384. 37946218 10.1186/s12916-023-03055-7 PMC10637015

[30] Celis-Morales CA, Lyall DM, Welsh P, Anderson J, Steell L, Guo Y, et al. Association between active commuting and incident cardiovascular disease, cancer, and mortality: prospective cohort study. BMJ (Clinical research ed). 2017;357:j1456. 28424154 10.1136/bmj.j1456

[31] Pearce M, Strain T, Wijndaele K, Sharp SJ, Mok A, Brage S. Is occupational physical activity associated with mortality in UK Biobank? Int J Behav Nutr Phys Act. 2021;18(1):102. 34315448 10.1186/s12966-021-01154-3 PMC8314512

[32] Bush K, Wilkinson T, Schnier C, Nolan J, Sudlow CLM. Definitions of dementia and the major diagnostic pathologies, UK Biobank Phase 1 outcomes adjudication 2018. Available from: https://biobank.ctsu.ox.ac.uk/crystal/crystal/docs/alg_outcome_dementia.pdf.

[33] Welmer A-K, Wang R, Rizzuto D, Ek S, Vetrano DL, Qiu C. Associations of blood pressure with risk of injurious falls in old age vary by functional status: A cohort study. Exp Gerontol. 2020;140:111038. 32738383 10.1016/j.exger.2020.111038

[34] Kongsted A, Nielsen AM. Latent class analysis in health research. J Physiother. 2017;63(1):55–8. 27914733 10.1016/j.jphys.2016.05.018

[35] Wang R, Marseglia A, Skoog J, Lindberg O, Pereira JB, Shams S, et al. Neuroimaging correlates of 3 distinct physical-cognitive phenotypes in cognitively normal older adults: The Gothenburg H70 cohort study. Neurology. 2025;104(1):e210121. 39642342 10.1212/WNL.0000000000210121 PMC11627174

[36] Meira-Machado L, Uña-Alvarez JD, Cadarso-Suárez C, Andersen PK. Multi-state models for the analysis of time-to-event data. Stat Methods Med Res. 2009;18(2):195–222. 18562394 10.1177/0962280208092301 PMC2692556

[37] Hu X, Wang J, Yang T, Jin J, Zeng Q, Aboubakri O, et al. Role of residential greenspace in the trajectory of major neurological disorders: A longitudinal study in UK Biobank. Sci Total Environ. 2024;912:168967. 38042194 10.1016/j.scitotenv.2023.168967

[38] Lange-Maia BS, Wagner M, Rogers CA, Mehta RI, Bennett DA, Tangney C, et al. Profiles of lifestyle health behaviors and postmortem dementia-related neuropathology. J Gerontol A Biol Sci Med Sci. 2024;79(5):glae100. 38597160 10.1093/gerona/glae100 PMC11059256

[39] Burstrom B, Whitehead M, Lindholm C, Diderichsen F. Inequality in the social consequences of illness: how well do people with long-term illness fare in the British and Swedish labor markets? Int J Health Serv. 2000;30(3):435–51. 11109175 10.2190/6PP1-TDEQ-H44D-4LJQ

[40] Alavinia SM, Burdorf A. Unemployment and retirement and ill-health: a cross-sectional analysis across European countries. Int Arch Occup Environ Health. 2008;82(1):39–45. 18264715 10.1007/s00420-008-0304-6 PMC2467501

[41] Yuan M, Xu C, Fang Y. The transitions and predictors of cognitive frailty with multi-state Markov model: a cohort study. BMC Geriatr. 2022;22(1):550. 35778705 10.1186/s12877-022-03220-2 PMC9248089

[42] Liu J, Wu Y, Long Z, Zhang S, Wu S. The association between cognitive frailty and the risk of fall occurrence in older adults: a meta-analysis of cohort studies. Front Med (Lausanne). 2025;12:1537240. 40012978 10.3389/fmed.2025.1537240 PMC11861544

[43] Zhong YJ, Meng Q, Su CH. Mechanism-driven strategies for reducing fall risk in the elderly: a multidisciplinary review of exercise interventions. Healthcare (Basel). 2024;12(23):2394. 39685016 10.3390/healthcare12232394 PMC11641686

[44] Aflaki K, Vigod S, Ray JG. Part I: A friendly introduction to latent class analysis. J Clin Epidemiol. 2022;147:168–70. 35636591 10.1016/j.jclinepi.2022.05.008

[45] Fry A, Littlejohns TJ, Sudlow C, Doherty N, Adamska L, Sprosen T, et al. Comparison of sociodemographic and health-related characteristics of UK biobank participants with those of the general population. Am J Epidemiol. 2017;186(9):1026–34. 28641372 10.1093/aje/kwx246 PMC5860371

