# Supplementary material for: Activity profiles and their associations with transitions between fall and dementia: findings from a multistate time-to-event analysis
Source: Alzheimers Res Ther. 2026 Jul 10;18:162. doi: 10.1186/s13195-026-02140-2 (PMC13366702; doi:10.1186/s13195-026-02140-2)
Supplement: Supplementary file 1 — Supplementary Material 1. [file 13195_2026_2140_MOESM1_ESM.docx]

**Supplementary Materials**

Supplemental Method

S-Figure 1: Flow chart of study participants

S-Figure 2: Directed acyclic graph illustrating the assumed relationships between covariates (white), exposures (green), and outcomes (blue).

S-Table 1. Parameters for model fit and diagnostics

S-Table 2. Baseline characteristics of study participants by activity profile groups

S-Table 3. Hazard ratios (HRs) and 95% confidence intervals (CIs) for multistate transitions across health, fall, dementia, and death according to defining components for the four individual activity factors among non-working groups

S-Table 4. Hazard ratios (HRs) and 95% confidence intervals (CIs) for multistate transitions across health, fall, dementia, and death by activity profile in the full sample and in non-working groups, excluding incident fall and dementia within three years after baseline

S-Table 5. Hazard ratios (HRs) and 95% confidence intervals (CIs) for multistate transitions across health, fall, dementia, and death by activity profile in the full sample and in non-working groups, excluding incident fall and dementia within ten years after baseline

S-Table 6. Physical activity and social isolation in non-working individuals by multistate transitions, excluding falls and dementia within three years of baseline

S-Table 7. Physical activity and social isolation in non-working individuals by multistate transitions, excluding falls and dementia within ten years of baselineS-Table 8. Activity profile membership by transitions in full and stratified samples (working vs. non-working), with additional covariates included

S-Table 9. Physical activity and social isolation in non-working individuals by transitions, with additional covariates included

S-Table 10. Activity profile membership and transitions in the full sample, non-working sub-group, with non-retired/non-working individuals excluded

S-Table 11. Activity profile components and transitions in the non-working sub-group, with non-retired/non-working individuals excluded

S-Table 12. Hazard ratios (HRs) and 95% confidence intervals (CIs) for multistate transitions across health, fall, dementia, and death by activity profile using posterior probabilities

**Supplemental Method:**

*UK Biobank field-IDs of exposure and outcome variables:*

Physical activity: 22038, 22029

Transport activity: 6143, 6142

Occupational activity: 816

Sleep quality: 1180, 1160, 1200, 1210, 1220

Social activity: 2110, 2020, 1031,6160, 709

All-cause dementia: 42018–42025, 131036–1310343, 40001, 40002, 41270, 41271, 41280, 41281, 42040

Injurious fall: 41270, 41271

Death: 40000

*Assessment of covariates*

Age at baseline was calculated by subtracting the participant’s birthdate from their baseline assessment date. Biological sex was classified as female or male. Education levels were grouped into three categories: college or university degree or higher, no college or university degree, and unknown.^1^ To adjust for chronic health conditions at baseline, we computed the Charlson Comorbidities Index (CCI) for each participant. The CCI consists here of 15 comorbidities (usually 16, but dementia was excluded here), which are weighted with 1 to 6 points, based on disease severity, resulting in a total score ranging from 0 to 32 points.^2,3^ If comorbidities were registered before the participant’s baseline assessment, the corresponding score was assigned. Vision problems were considered as present, if a participant self-reported any eye problems/disorders or other eye problems. Hearing problems were categorized into ‘present’, ‘not present’, and ‘with hearing aid’. Grip strength, measured in kilograms, was calculated as the sum of left and right handgrip strength. A dichotomous heavy drinking variable (yes/no) was created by converting participants’ self-reported weekly alcohol consumption into standard UK alcohol units using guidelines provided by the National Health Service. Heavy drinking was defined as >35 units/week for women and >50 units/week for men.^4^ Apolipoprotein E (APOE) genotype status was determined for each participant. Individuals with one or two APOE ε4 alleles were classified as APOE ε4 carriers, while those without APOE ε4 alleles were classified as APOE ε4 non-carriers.

In the multistate models, all transitions were adjusted for age, sex, education, Charlson Comorbidity Index (CCI), hearing, vision, handgrip strength and heavy drinking, while transitions involving dementia were additionally adjusted for APOE ε4 status.


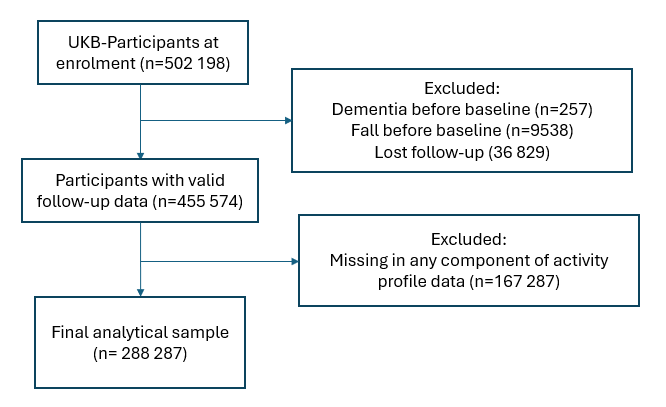


**S-Figure 1**: Flow chart of study participants


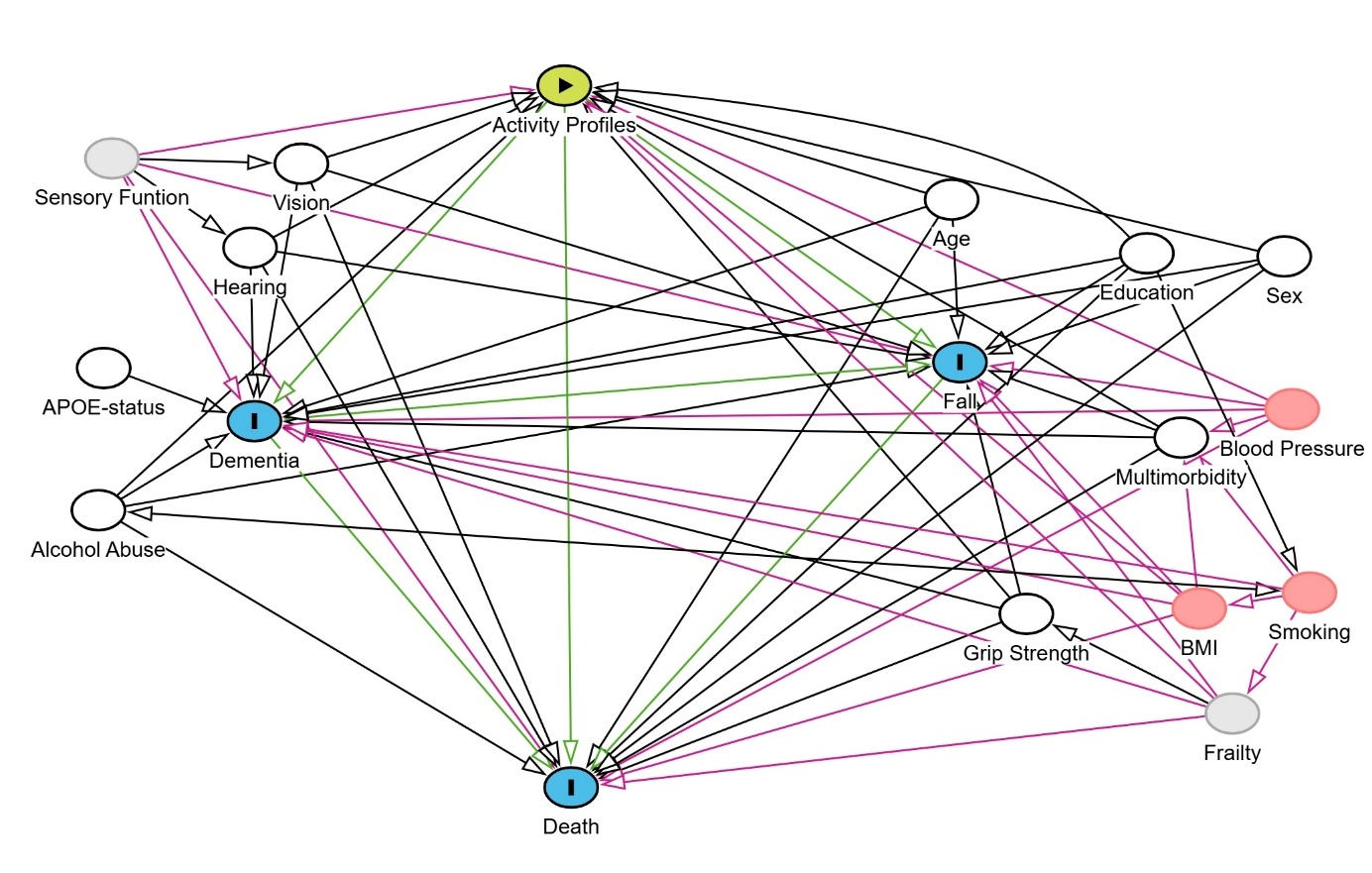


**S-Figure 2**: Directed acyclic graph illustrating the assumed relationships between exposures (green), and outcomes (blue).

*Notes*. White nodes represent measured covariates and confounders that were adjusted for in the primary model. Red nodes represent distal ancestors of these confounders or indirect confounders; these were not included in the primary adjustment set but were incorporated in sensitivity analyses to assess the robustness of our findings. Grey nodes represent latent or unobserved variables that could not be directly measured in the current study.

*Distal ancestors* are upstream variables in a causal pathway that influence the exposure and/or outcome indirectly through more proximal confounders.

**S-Table 1.** Parameters for model fit and diagnostics

|  | **Model fit criteria** | | | | | | **Diagnostic criteria** | | | | | | | |
| --- | --- | --- | --- | --- | --- | --- | --- | --- | --- | --- | --- | --- | --- | --- |
| Models | LL(model) | df | AIC | ∆AIC% | BIC | ∆BIC% | Smallest class size (%) | Entropy | ALCPP | | | | | |
|  |  |  |  |  |  |  |  |  | Class 1 | Class 2 | Class 3 | Class 4 | Class 5 | Class 6 |
| 2 Class | -1 445 076 | 1 | 2 890 207 | - | 2 890 492 | - | 43.5% | 1 | 1 | 1 |  |  |  |  |
| 3 Class | -1 441 288 | 1 | 2 882 658 | 7549 | 2 883 092 | 7400 | 14.4% | 0.79 | 0.73 | 1 | 0.80 |  |  |  |
| 4 Class | -1 433 221 | 1 | 2 866 551 | **16 107** | 2 867 133 | **15 959** | 14.4% | **0.8** | 0.80 | **0.73** | 0.92 | 0.95 |  |  |
| 5 Class | -1 430 710 | 1 | **2 861 559** | 4992 | **2 862 288** | 4845 | 11.7% | 0.7 | 0.81 | 0.81 | 0.73 | 0.80 | 0.64 |  |
| 6 Class | -1 433 499 | 1 | 2 867 164 | -5605 | 2 868 042 | -5754 | 3.2% | 0.73 | 0.69 | 1 | 0.52 | 0.84 | 0.62 | 0.69 |

***Note***: Bold text indicates that the model met optimal criteria or was preferred over other models. We chose the 4-class model based on several criteria: reduction in AIC and BIC, smallest class size (>10%), entropy (>0.8),^5,6^ and average latent class posterior probability (≥0.7 for each class).^5,7^

Abbreviations: LL=Log-likelihood; AIC=Akaike information criterion; BIC=Bayesian information criterion; ALCPP=average latent class posterior probability.

**S-Table 2.** Baseline characteristics of study participants by activity profile groups

| **Characteristics** | **Active non-working profile** | **Inactive, non-working profile** | **Active working profile** | **Inactive, working profile** | **P-value** |
| --- | --- | --- | --- | --- | --- |
|  | (n=84066) | (n=41415) | (n=77097) | (n=85709) |  |
| **Age** (years), median (IQR) | 63.9 (60.6 to 66.8) | 63.1 (58.7 to 66.5) | 52.6 (46.9 to 58.5) | 53.3 (47.5 to 58.6) | <0.001 |
| **Sex**, n (%) |  |  |  |  |  |
| Women | 47301 (56.3) | 22459 (54.2) | 37367 (48.5) | 47507 (55.4) | <0.001 |
| Men | 36765 (43.7) | 18956 (45.8) | 39 730 (51.5) | 38202 (44.6) |  |
| **Education**, n (%) |  |  |  |  |  |
| University or higher | 24797 (29.5) | 9119 (22.0) | 27761 (36.0) | 36817 (43.0) | <0.001 |
| Below University | 58592 (69.7) | 31906 (77.0) | 49040 (63.6) | 48671 (56.8) |  |
| Unknown | 677 (0.8) | 390 (0.9) | 296 (0.4) | 221 (0.3) |  |
| ***APOE* ε4 status**, n (%) |  |  |  |  |  |
| ε4 carrier | 19871 (23.6) | 9502 (22.9) | 18456 (23.9) | 20228 (23.6) | <0.001 |
| Non-ε4 carrier | 49902 (59.4) | 23977 (57.9) | 45608 (59.2) | 51004 (59.5) |  |
| Missing | 14293 (17.0) | 7936 (19.2) | 13033 (16.9) | 14477 (16.9) |  |
| **Smoking** **status**, n (%) |  |  |  |  |  |
| Current | 5830 (6.9) | 6321 (15.3) | 8109 (10.5) | 8783 (10.2) | <0.001 |
| Never | 44145 (52.5) | 18663 (45.1) | 43347 (56.2) | 49604 (57.9) |  |
| Previous | 33881 (40.3) | 16272 (39.3) | 25514 (33.1) | 27174 (31.7) |  |
| Missing | 210 (0.2) | 159 (0.4) | 127 (0.2) | 148 (0.2) |  |
| **BMI status**, n (%) |  |  |  |  |  |
| Underweight (<18.5) | 405 (0.5) | 283 (0.7) | 347 (0.5) | 374 (0.4) | <0.001 |
| Normal (18.5≤BMI<25) | 27900 (33.2) | 10768 (26.0) | 28367 (36.8) | 27132 (31.7) |  |
| Overweight (25≤BMI<30) | 37713 (44.9) | 16778 (40.5) | 33046 (42.9) | 35691 (41.6) |  |
| Obese (≥30) | 17678 (21.0) | 13209 (31.9) | 15080 (19.6) | 22221 (25.9) |  |
| Missing | 370 (0.4) | 377 (0.9) | 257 (0.3) | 291 (0.3) |  |
| **SBP** (mmHg), mean (SD) | 142.2 (19.0) | 140.5 (19.0) | 134.8 (17.7) | 134.3 (17.7) | <0.001 |
| **Grip strength** (kilograms), mean (SD) | 58.2 (20.6) | 55.8 (21.3) | 67.5 (22.2) | 63.6 (21.6) | <0.001 |
| **Vision problem**, n (%) |  |  |  |  |  |
| Yes | 14975 (17.8) | 9218 (22.3) | 9489 (12.3) | 10960 (12.8) | <0.001 |
| No | 68739 (81.8) | 31911 (77.1) | 67342 (87.3) | 74491 (86.9) |  |
| Missing | 352 (0.4) | 286 (0.7) | 266 (0.3) | 258 (0.3) |  |
| **Hearing problem/disorder**, n (%) | |  |  |  |  |
| Yes | 20277 (24.1) | 10850 (26.2) | 15551 (20.2) | 17428 (20.3) | <0.001 |
| Yes, with hearing aid | 3920 (4.7) | 2072 (5.0) | 1235 (1.6) | 1403 (1.6) |  |
| No | 57269 (68.1) | 27014 (65.2) | 57771 (74.9) | 64141 (74.8) |  |
| Missing | 2600 (3.1) | 1479 (3.6) | 2540 (3.3) | 2737 (3.2) |  |
| **Physical activity categories**, n (%) | |  |  |  |  |
| <600 METs | 18282 (21.7) | 28066 (67.8) | 0 (0.0) | 67253 (78.5) | <0.001 |
| 600-1200 METs | 16928 (20.1) | 5066 (12.2) | 10547 (13.7) | 18456 (21.5) |  |
| >1200 METs | 48856 (58.1) | 8283 (20.0) | 66550 (86.3) | 0 (0.0) |  |
| Sleep score, n (%) |  |  |  |  |  |
| 0 | 0 (0.0) | 427 (1.0) | 109 (0.1) | 143 (0.2) | <0.001 |
| 1 | 1992 (2.4) | 3732 (9.0) | 2641 (3.4) | 3767 (4.4) |  |
| 2 | 14447 (17.2) | 11132 (26.9) | 13717 (17.8) | 17404 (20.3) |  |
| 3 | 33579 (39.9) | 15229 (36.8) | 28846 (37.4) | 32630 (38.1) |  |
| 4 | 27266 (32.4) | 10085 (24.4) | 24821 (32.2) | 25556 (29.8) |  |
| 5 | 6782 (8.1) | 810 (2.0) | 6963 (9.0) | 6209 (7.2) |  |
| **Functional Isolation**, n (%) |  |  |  |  |  |
| Present | 8635 (10.3) | 25913 (62.6) | 18944 (24.6) | 21977 (25.6) | <0.001 |
| Not present | 75431 (89.7) | 15502 (37.4) | 58153 (75.4) | 63732 (74.4) |  |
| **Structural Isolation**, n (%) |  |  |  |  |  |
| Present | 19083 (22.7) | 35092 (84.7) | 29809 (38.7) | 44189 (51.6) | <0.001 |
| Not present | 64983 (77.3) | 6323 (15.3) | 47288 (61.3) | 41520 (48.4) |  |
| **Transport Activity**, n (%) |  |  |  |  |  |
| Not working | 84066 (100.0) | 41415 (100.0) | 0 (0.0) | 0 (0.0) | <0.001 |
| Non-active | 0 (0.0) | 0 (0.0) | 55070 (71.4) | 71904 (83.9) |  |
| Active | 0 (0.0) | 0 (0.0) | 22027 (28.6) | 13805 (16.1) |  |
| **Occupational Activity**, n (%) |  |  |  |  |  |
| Not working | 84 066 (100.0) | 41415 (100.0) | 0 (0.0) | 0 (0.0) | <0.001 |
| No manual | 0 (0.0) | 0 (0.0) | 39325 (51.0) | 73435 (85.7) |  |
| Manual | 0 (0.0) | 0 (0.0) | 37772 (49.0) | 12274 (14.3) |  |

**Abbreviations.**

IQR = Interquartile Range; SD = Standard Deviation; BMI = body mass index; MET = Metabolic Equivalent of Task

*Notes*. Physical activity categories were defined based on weekly moderate-to-vigorous physical activity, expressed in metabolic equivalent of task (MET) units, and classified according to the World Health Organization’s weekly physical activity recommendations.

**S-Table 3.** Hazard ratios (HRs) and 95% confidence intervals (CIs) for multistate transitions across health, fall, dementia, and death according to defining components for the four individual activity factors among non-working groups

| **Non-working individuals (n=125 481)** | | | | | | | | |
| --- | --- | --- | --- | --- | --- | --- | --- | --- |
|  | **Healthy to fall (n=12 073)^a^** | **Healthy to dementia (n=3515)^b^** | **Healthy to death (n=12 901)^a^** | **Fall to fall & dementia (n=771)^b^** | **Fall to death (n=2391)^a^** | **Dementia to fall & dementia (n=964)^b^** | **Dementia to death (n=1314)^b^** | **Fall & dementia to death (n=962)^b^** |
|  | HRs (95% CI) | HRs (95% CI) | HRs (95% CI) | HRs (95% CI) | HRs (95% CI) | HRs (95% CI) | HRs (95% CI) | HRs (95% CI) |
| **Defining components for physical activity** | | | | | | | | |
| *Moderate Physical activity* | | | | | | | | |
| <600 MET-min/week | Ref. | Ref. | Ref. | Ref. | Ref. | Ref. | Ref. | Ref. |
| 600-1200 MET-min/week | 0.88  (0.84, 0.92)^***^ | 0.90  (0.83, 0.99)^*^ | 0.84  (0.80, 0.88)^***^ | 0.84  (0.69, 1.03) | 0.80  (0.71, 0.90)^***^ | 0.93  (0.78, 1.11) | 0.98  (0.84, 1.14) | 1.02  (0.85, 1.22) |
| >1200 MET-min/week | 0.91  (0.87, 0.94)^***^ | 0.92  (0.86, 0.99)^*^ | 0.84  (0.80, 0.87)^***^ | 0.92  (0.78, 1.09) | 0.89  (0.81, 0.98)^*^ | 0.86  (0.74, 0.99)^*^ | 0.96  (0.85, 1.09) | 0.91  (0.78, 1.05) |
| *Vigorous Physical activity* | | | | | | | | |
| <600 MET-min/week | Ref. | Ref. | Ref. | Ref. | Ref. | Ref. | Ref. | Ref. |
| 600-1200 MET-min/week | 0.90  (0.85, 0.95)^***^ | 0.95  (0.86, 1.06) | 0.79  (0.75, 0.83)^***^ | 0.77  (0.61, 0.99)^*^ | 0.84  (0.74, 0.96)^*^ | 0.67  (0.54, 0.83)^***^ | 0.95  (0.81, 1.12) | 0.91  (0.73, 1.13) |
| >1200 MET-min/week | 0.88  (0.83, 0.92)^***^ | 1.00  (0.92, 1.10) | 0.77  (0.73, 0.81)^***^ | 0.92  (0.74, 1.13) | 0.80  (0.70, 0.90)^***^ | 0.94  (0.79, 1.12) | 0.92  (0.79, 1.07) | 0.96  (0.80, 1.14) |
| **Defining components for structural isolation** | | | | | | | | |
| *Friend/family visits/contacts* | | | | | | | | |
| Never or almost never | Ref. | Ref. | Ref. | Ref. | Ref. | Ref. | Ref. | Ref. |
| Once every 3 months | 0.70  (0.61, 0.81)^***^ | 0.77  (0.59, 1.00) | 0.74  (0.65, 0.84)^***^ | 0.98  (0.62, 1.56) | 0.84  (0.65, 1.10) | 1.27  (0.74, 2.16) | 0.83  (0.54, 1.27) | 2.00  (1.24, 3.25)^**^ |
| Monthly | 0.65  (0.57, 0.74)^***^ | 0.66  (0.51, 0.85)^**^ | 0.63  (0.56, 0.71)^***^ | 0.76  (0.49, 1.18) | 0.92  (0.73, 1.17) | 1.37  (0.83, 2.28) | 1.09  (0.73, 1.61) | 2.02  (1.26, 3.22)^**^ |
| Weekly | 0.61  (0.53, 0.69)^***^ | 0.60  (0.47, 0.75)^***^ | 0.61  (0.54, 0.68)^***^ | 0.71  (0.48, 1.07) | 0.83  (0.67, 1.04) | 1.23  (0.76, 1.98) | 0.98  (0.68, 1.42) | 1.89  (1.22, 2.93)^**^ |
| 2-4 times a week | 0.59  (0.52, 0.67)^***^ | 0.66  (0.52, 0.83)^***^ | 0.61  (0.55, 0.69)^***^ | 0.76  (0.51, 1.13) | 0.72  (0.58, 0.90)^**^ | 1.24  (0.77, 1.99) | 1.01  (0.70, 1.45) | 1.75  (1.13, 2.71)^*^ |
| Daily | 0.65  (0.57, 0.74)^***^ | 0.66  (0.52, 0.85)^***^ | 0.62  (0.55, 0.70)^***^ | 0.68  (0.44, 1.04) | 0.73  (0.58, 0.92)^**^ | 1.36  (0.83, 2.21) | 1.01  (0.69, 1.48) | 1.49  (0.94, 2.35) |
| Missing | 0.55  (0.36, 0.82)^**^ | 0.64  (0.33, 1.24) | 0.84  (0.62, 1.15) | 0.78  (0.18, 3.27) | 0.47  (0.17, 1.28) | 2.42  (0.81, 7.20) | 2.91  (1.12, 7.54)^*^ | 0.51  (0.12, 2.21) |
| *No weekly group activities* | 1.00  (0.96, 1.05) | 1.18  (1.09, 1.27)^***^ | 1.20  (1.15, 1.24)^***^ | 1.09  (0.93, 1.28) | 1.27  (1.17, 1.39)^***^ | 1.03  (0.90, 1.19) | 1.10  (0.98, 1.24) | 0.94  (0.81, 1.08) |
| *Living alone* | 1.45  (1.39, 1.51)^***^ | 1.16  (1.07, 1.26)^***^ | 1.38  (1.32, 1.44)^***^ | 1.32  (1.13, 1.54)^***^ | 1.24  (1.13, 1.35)^***^ | 1.09  (0.94, 1.28) | 0.92  (0.80, 1.06) | 0.83  (0.72, 0.96)^*^ |
| **Defining components for functional social isolation** | | | | | | | | |
| *Ability to confide* |  |  |  |  |  |  |  |  |
| Never or almost never | Ref. | Ref. | Ref. | Ref. | Ref. | Ref. | Ref. | Ref. |
| Once every few months | 0.98  (0.90, 1.06) | 0.97  (0.84, 1.13) | 0.90  (0.83, 0.98)^*^ | 0.99  (0.72, 1.37) | 0.86  (0.71, 1.03) | 1.26  (0.97, 1.64) | 1.08  (0.83, 1.40) | 0.84  (0.64, 1.10) |
| About once a month | 0.98  (0.89, 1.06) | 1.06  (0.91, 1.24) | 0.86  (0.79, 0.94)^***^ | 1.15  (0.83, 1.59) | 1.12  (0.93, 1.35) | 0.95  (0.71, 1.27) | 1.19  (0.93, 1.52) | 0.84  (0.63, 1.13) |
| About once a week | 0.92  (0.86, 0.99)^*^ | 0.95  (0.84, 1.08) | 0.90  (0.85, 0.97)^**^ | 1.05  (0.80, 1.37) | 1.03  (0.89, 1.20) | 0.81  (0.64, 1.03) | 0.83  (0.67, 1.04) | 0.82  (0.64, 1.04) |
| 2-4 times a week | 0.98  (0.91, 1.05) | 0.85  (0.74, 0.98)^*^ | 0.86  (0.80, 0.93)^***^ | 1.26  (0.95, 1.66) | 0.96  (0.81, 1.14) | 0.86  (0.66, 1.12) | 0.90  (0.70, 1.14) | 0.96  (0.73, 1.25) |
| Daily or almost daily | 0.85  (0.81, 0.90)^***^ | 0.77  (0.70, 0.84)^***^ | 0.83  (0.79, 0.87)^***^ | 0.88  (0.72, 1.07) | 0.94  (0.84, 1.05) | 0.82  (0.69, 0.98)^*^ | 1.17  (1.01, 1.37)^*^ | 1.07  (0.90, 1.26) |
| Missing | 0.93  (0.82, 1.05) | 1.05  (0.86, 1.28) | 0.89  (0.79, 1.00)^*^ | 1.12  (0.73, 1.72) | 0.69  (0.51, 0.92)^*^ | 1.23  (0.87, 1.75) | 0.94  (0.66, 1.34) | 1.01  (0.70, 1.45) |
| *Frequent feelings of loneliness* | | | | | | | | |
| No | Ref. | Ref. | Ref. | Ref. | Ref. | Ref. | Ref. | Ref. |
| Yes | 1.34  (1.29, 1.41)^***^ | 1.31  (1.20, 1.43)^***^ | 1.21  (1.15, 1.26)^***^ | 1.45  (1.23, 1.71)^***^ | 1.15  (1.05, 1.27)^**^ | 0.89  (0.76, 1.05) | 0.85  (0.73, 0.98)^*^ | 0.95  (0.81, 1.11) |
| **Defining components for sleep score** | | | | | | | | |
| *Early chronotype* | 0.89  (0.86, 0.92)^***^ | 0.96  (0.90, 1.03) | 0.88  (0.85, 0.91)^***^ | 0.96  (0.82, 1.11) | 0.87  (0.80, 0.95)^**^ | 1.01  (0.88, 1.16) | 1.05  (0.94, 1.18) | 1.11  (0.97, 1.27) |
| *7—8 hours of sleep per day vs. other durations* | 0.83  (0.80, 0.86)^***^ | 0.90  (0.84, 0.97)^**^ | 0.84  (0.81, 0.87)^***^ | 0.80  (0.69, 0.92)^**^ | 0.85  (0.79, 0.93)^***^ | 1.01  (0.88, 1.15) | 1.02  (0.91, 1.14) | 1.25  (1.09, 1.43)^***^ |
| *No or rare insomnia* | 0.97  (0.92, 1.02) | 1.31  (1.21, 1.42)^***^ | 0.96  (0.92, 1.00) | 1.10  (0.92, 1.31) | 0.98  (0.88, 1.09) | 0.84  (0.72, 0.98)^*^ | 1.14  (1.01, 1.28)^*^ | 0.95  (0.82, 1.11) |
| *Absence of snoring* | 1.09  (1.05, 1.13)^***^ | 1.05  (0.98, 1.13) | 1.08  (1.04, 1.12)^***^ | 1.15  (0.99, 1.35) | 1.10  (1.01, 1.20)^*^ | 1.05  (0.91, 1.20) | 1.04  (0.93, 1.17) | 0.98  (0.86, 1.13) |
| *No frequent daytime sleepiness* | 0.80  (0.74, 0.87)^***^ | 0.83  (0.71, 0.97)^*^ | 0.90  (0.83, 0.97)^**^ | 0.79  (0.59, 1.06) | 0.84  (0.71, 0.99)^*^ | 1.00  (0.74, 1.35) | 0.94  (0.72, 1.21) | 1.00  (0.75, 1.32) |

^a^Model was adjusted for age, sex, education, multimorbidity, hearing, vision, handgrip strength and heavy drinking.

^b^Model was adjusted for age, sex, education, multimorbidity, hearing, vision, handgrip strength, heavy drinking, and presence of APOE4-allele

HR=hazard ratio; CI=confidence interval; PA = physical activity; MET = metabolic task equivalent.

^*^P<0.05; ^**^P<0.01; ^***^P<0.001

**S-Table 4.** Hazard ratios (HRs) and 95% confidence intervals (CIs) for multistate transitions across health, fall, dementia, and death by activity profile in the full sample and in non-working groups, excluding incident fall and dementia within three years after baseline

| Activity profile membership by transition in full sample (n=285 068) | | | | | | | | |
| --- | --- | --- | --- | --- | --- | --- | --- | --- |
|  | **Healthy to fall (n=16 689)^a^** | **Healthy to dementia (n=4252)^b^** | **Healthy to death (n=19 723)^a^** | **Fall to fall & dementia (n=739)^b^** | **Fall to death (n=2598)^a^** | **Dementia to fall & dementia (n=1110)^b^** | **Dementia to death (n=1526)^b^** | **Fall & dementia to death (n=992)^b^** |
|  | HRs (95% CI) | HRs (95% CI) | HRs (95% CI) | HRs (95% CI) | HRs (95% CI) | HRs (95% CI) | HRs (95% CI) | HRs (95% CI) |
| Active, working profile | Ref. | Ref. | Ref. | Ref. | Ref. | Ref. | Ref. | Ref. |
| Non-active, working profile | 1.02 (0.97, 1.07) | 1.02 (0.89, 1.16) | 1.03 (0.98, 1.08) | 1.38 (0.94, 2.01) | 1.07 (0.92, 1.25) | 0.92 (0.69, 1.23) | 1.08 (0.86, 1.36) | 1.17 (0.83, 1.6 |
| Active, non-working profile | 1.11 (1.06, 1.17)^***^ | 1.34 (1.20, 1.51)^***^ | 1.15 (1.10, 1.20)^***^ | 1.71 (1.23, 2.37)^**^ | 1.14 (0.99, 1.32) | 1.03 (0.82, 1.31) | 1.10 (0.91, 1.34) | 1.14 (0.86, 1.50) |
| Non-active, non-working profile | 1.45 (1.37, 1.52)^***^ | 1.64 (1.46, 1.85)^***^ | 1.62 (1.54, 1.70)^***^ | 2.14 (1.53, 2.98)^***^ | 1.55 (1.34, 1.79)^***^ | 1.21 (0.95, 1.55) | 1.03 (0.84, 1.27) | 1.15 (0.87, 1.52) |
| Activity profile membership by transition in non-working profiles (n=123 523) | | | | | | | | |
|  | **Healthy to fall (n=10 255)^a^** | **Healthy to dementia (n=3375)^b^** | **Healthy to death (n=12 901)^a^** | **Fall to fall & dementia (n=626)^b^** | **Fall to death (n=1955)^a^** | **Dementia to fall & dementia (n=929)^b^** | **Dementia to death (n=1238)^b^** | **Fall & dementia to death (n=852)^b^** |
|  | HRs (95% CI) | HRs (95% CI) | HRs (95% CI) | HRs (95% CI) | HRs (95% CI) | HRs (95% CI) | HRs (95% CI) | HRs (95% CI) |
| Active, non-working profile | Ref. | Ref. | Ref. | Ref. | Ref. | Ref. | Ref. | Ref. |
| Non-active, non-working profile | 1.29 (1.24, 1.34)^***^ | 1.23 (1.14, 1.32)^***^ | 1.39 (1.34, 1.44)^***^ | 1.26 (1.07, 1.48)^**^ | 1.35 (1.23, 1.48)^***^ | 1.18 (1.03, 1.36)^*^ | 0.93 (0.83, 1.06) | 1.02 (0.89, 1.17) |

^a^Model was adjusted for age, sex, education, Charlson Comorbidity Index, hearing, vision, handgrip strength and heavy drinking.

^b^Model was adjusted for age, sex, education, Charlson Comorbidity Index, hearing, vision, handgrip strength, heavy drinking, and presence of APOE4-allele

HR=hazard ratio; CI=confidence interval.

^*^P<0.05; ^**^P<0.01; ^***^P<0.001

**S-Table 5.** Hazard ratios (HRs) and 95% confidence intervals (CIs) for multistate transitions across health, fall, dementia, and death by activity profile in the full sample and in non-working groups, excluding incident fall and dementia within ten years after baseline

| Activity profile membership by transition in full sample (n=273 656) | | | | | | | | |
| --- | --- | --- | --- | --- | --- | --- | --- | --- |
|  | **Healthy to fall (n=7178)^a^** | **Healthy to dementia (n=2351)^b^** | **Healthy to death (n=19 723)^a^** | **Fall to fall & dementia (n=227)^b^** | **Fall to death (n=929)^a^** | **Dementia to fall & dementia (n=580)^b^** | **Dementia to death (n=662)^b^** | **Fall & dementia to death (n=331)^b^** |
|  | HRs (95% CI) | HRs (95% CI) | HRs (95% CI) | HRs (95% CI) | HRs (95% CI) | HRs (95% CI) | HRs (95% CI) | HRs (95% CI) |
| Active, working profile | Ref. | Ref. | Ref. | Ref. | Ref. | Ref. | Ref. | Ref. |
| Non-active, working profile | 1.03 (0.96, 1.11) | 0.93 (0.78, 1.11) | 1.03 (0.98, 1.08) | 1.58 (0.80, 3.11) | 1.20 (0.92, 1.55) | 0.93 (0.62, 1.39) | 1.08 (0.77, 1.52) | 1.08 (0.60, 1.94) |
| Active, non-working profile | 1.15 (1.07, 1.24)^***^ | 1.22 (1.05, 1.42)^**^ | 1.15 (1.10, 1.21)^***^ | 1.76 (0.96, 3.22) | 1.15 (0.90, 1.47) | 0.93 (0.67, 1.29) | 0.94 (0.71, 1.26) | 1.45 (0.90, 2.34) |
| Non-active, non-working profile | 1.47 (1.36, 1.60)^***^ | 1.44 (1.23, 1.69)^***^ | 1.63 (1.56, 1.71)^***^ | 1.95 (1.05, 3.62)^*^ | 1.41 (1.10, 1.80)^**^ | 1.09 (0.77, 1.53) | 0.97 (0.72, 1.32) | 1.49 (0.91, 2.43) |
| Activity profile membership by transition in non-working profiles (n=116 232) | | | | | | | | |
|  | **Healthy to fall (n=4491)^a^** | **Healthy to dementia (n=1848)^b^** | **Healthy to death (n=12 901)^a^** | **Fall to fall & dementia (n=190)^b^** | **Fall to death (n=697)^a^** | **Dementia to fall & dementia (n=481)^b^** | **Dementia to death (n=387)^b^** | **Fall & dementia to death (n=284)^b^** |
|  | HRs (95% CI) | HRs (95% CI) | HRs (95% CI) | HRs (95% CI) | HRs (95% CI) | HRs (95% CI) | HRs (95% CI) | HRs (95% CI) |
| Active, non-working profile | Ref. | Ref. | Ref. | Ref. | Ref. | Ref. | Ref. | Ref. |
| Non-active, non-working profile | 1.26 (1.19, 1.34)^***^ | 1.18 (1.07, 1.30)^***^ | 1.40 (1.35, 1.45)^***^ | 1.10 (0.81, 1.49) | 1.24 (1.06, 1.45)^**^ | 1.18 (0.97, 1.44) | 1.02 (0.84, 1.23) | 1.06 (0.82, 1.36) |

^a^Model was adjusted for age, sex, education, Charlson Comorbidity Index, hearing, vision, handgrip strength and heavy drinking.

^b^Model was adjusted for age, sex, education, Charlson Comorbidity Index, hearing, vision, handgrip strength, heavy drinking, and presence of APOE4-allele

HR=hazard ratio; CI=confidence interval. ^*^P<0.05; ^**^P<0.01; ^***^P<0.001

**S-Table 6.** Physical activity and social isolation in non-working individuals by multistate transitions, excluding falls and dementia within three years of baseline

| Physical activity by transition in non-working individuals (n=123 523) | | | | | | | | |
| --- | --- | --- | --- | --- | --- | --- | --- | --- |
|  | **Healthy to fall (n=10 255)^a^** | **Healthy to dementia (n=3375)^b^** | **Healthy to death (n=12 901)^a^** | **Fall to fall & dementia (n=626)^b^** | **Fall to death (n=1955)^a^** | **Dementia to fall & dementia (n=929)^b^** | **Dementia to death (n=1238)^b^** | **Fall & dementia to death (n=852)^b^** |
| Walking |  |  |  |  |  |  |  |  |
| <600 MET-min/week | Ref. | Ref. | Ref. | Ref. | Ref. | Ref. | Ref. | Ref. |
| 600-1200 MET-min/week | 0.97 (0.92, 1.02) | 0.98 (0.90, 1.06) | 0.86 (0.82, 0.90)^***^ | 0.96 (0.79, 1.18) | 0.96 (0.86, 1.07) | 1.01 (0.86, 1.19) | 1.05 (0.91, 1.21) | 1.09 (0.92, 1.29) |
| >1200 MET-min/week | 0.95 (0.91, 0.99)^*^ | 0.98 (0.91, 1.06) | 0.85 (0.81, 0.88)^***^ | 0.95 (0.79, 1.14) | 0.89 (0.80, 0.99)^*^ | 0.86 (0.73, 1.00) | 1.01 (0.88, 1.15) | 0.93 (0.79, 1.10) |
| Moderate PA |  |  |  |  |  |  |  |  |
| <600 MET-min/week | Ref. | Ref. | Ref. | Ref. | Ref. | Ref. | Ref. | Ref. |
| 600-1200 MET-min/week | 0.87 (0.83, 0.92)^***^ | 0.92 (0.84, 1.01) | 0.84 (0.80, 0.88)^***^ | 0.85 (0.68, 1.07) | 0.82 (0.72, 0.93)^**^ | 0.91 (0.77, 1.09) | 0.98 (0.84, 1.14) | 0.99 (0.82, 1.20) |
| >1200 MET-min/week | 0.90 (0.86, 0.94)^***^ | 0.93 (0.86, 1.01) | 0.84 (0.80, 0.87)^***^ | 0.95 (0.79, 1.14) | 0.92 (0.83, 1.02) | 0.85 (0.73, 0.99)^*^ | 0.96 (0.85, 1.09) | 0.92 (0.79, 1.08) |
| Vigorous PA |  |  |  |  |  |  |  |  |
| <600 MET-min/week | Ref. | Ref. | Ref. | Ref. | Ref. | Ref. | Ref. | Ref. |
| 600-1200 MET-min/week | 0.91 (0.86, 0.96)^**^ | 0.95 (0.86, 1.06) | 0.79 (0.75, 0.83)^***^ | 0.76 (0.58, 0.99)^*^ | 0.84 (0.72, 0.97)^*^ | 0.67 (0.54, 0.84)^***^ | 0.98 (0.83, 1.15) | 0.91 (0.72, 1.16) |
| >1200 MET-min/week | 0.89 (0.84, 0.94)^***^ | 1.01 (0.92, 1.10) | 0.77 (0.73, 0.81)^***^ | 0.89 (0.71, 1.12) | 0.81 (0.70, 0.92)^**^ | 0.92 (0.77, 1.10) | 0.89 (0.76, 1.04) | 0.98 (0.81, 1.18) |
| Moderate-to-vigorous PA |  |  |  |  |  |  |  |  |
| <600 MET-min/week | Ref. | Ref. | Ref. | Ref. | Ref. | Ref. | Ref. | Ref. |
| 600-1200 MET-min/week | 0.85 (0.80, 0.90)^***^ | 0.90 (0.81, 0.99)^*^ | 0.79 (0.75, 0.84)^***^ | 0.92 (0.73, 1.15) | 0.78 (0.68, 0.89)^***^ | 0.83 (0.69, 1.01) | 0.97 (0.83, 1.14) | 1.19 (0.98, 1.45) |
| >1200 MET-min/week | 0.85 (0.81, 0.88)^***^ | 0.89 (0.83, 0.96)^**^ | 0.75 (0.73, 0.78)^***^ | 0.83 (0.69, 0.99)^*^ | 0.84 (0.76, 0.93)^***^ | 0.81 (0.70, 0.94)^**^ | 0.89 (0.78, 1.01) | 0.92 (0.79, 1.07) |

| Social isolation by transition in non-working individuals (n=123 523) | | | | | | | | |
| --- | --- | --- | --- | --- | --- | --- | --- | --- |
|  | **Healthy to fall (n=10 255)^a^** | **Healthy to dementia (n=3375)^b^** | **Healthy to death (n=12 901)^a^** | **Fall to fall & dementia (n=626)^b^** | **Fall to death (n=1955)^a^** | **Dementia to fall & dementia (n=929)^b^** | **Dementia to death (n=1238)^b^** | **Fall & dementia to death (n=852)^b^** |
|  | HRs (95% CI) | HRs (95% CI) | HRs (95% CI) | HRs (95% CI) | HRs (95% CI) | HRs (95% CI) | HRs (95% CI) | HRs (95% CI) |
| Ability to confide |  |  |  |  |  |  |  |  |
| Never or almost never | Ref. | Ref. | Ref. | Ref. | Ref. | Ref. | Ref. | Ref. |
| Once every few months | 1.01 (0.92, 1.10) | 0.96 (0.82, 1.12) | 0.90 (0.83, 0.98)^*^ | 1.15 (0.81, 1.64) | 0.76 (0.61, 0.94)^*^ | 1.38 (1.05, 1.80)^*^ | 1.20 (0.92, 1.58) | 0.83 (0.62, 1.11) |
| About once a month | 1.00 (0.91, 1.10) | 1.04 (0.89, 1.22) | 0.86 (0.79, 0.94)^***^ | 1.28 (0.89, 1.84) | 1.04 (0.85, 1.29) | 0.90 (0.67, 1.21) | 1.18 (0.91, 1.53) | 0.79 (0.58, 1.08) |
| About once a week | 0.96 (0.89, 1.03) | 0.94 (0.83, 1.07) | 0.90 (0.85, 0.97)^**^ | 1.22 (0.90, 1.64) | 0.98 (0.83, 1.15) | 0.83 (0.65, 1.06) | 0.87 (0.70, 1.10) | 0.80 (0.62, 1.03) |
| 2-4 times a week | 1.02 (0.94, 1.10) | 0.84 (0.72, 0.97)^*^ | 0.86 (0.80, 0.93)^***^ | 1.41 (1.03, 1.92)^*^ | 0.94 (0.78, 1.13) | 0.90 (0.68, 1.18) | 0.97 (0.76, 1.26) | 0.95 (0.71, 1.26) |
| Daily or almost daily | 0.89 (0.84, 0.94)^***^ | 0.77 (0.70, 0.85)^***^ | 0.83 (0.79, 0.87)^***^ | 0.96 (0.76, 1.20) | 0.91 (0.81, 1.03) | 0.84 (0.71, 1.01) | 1.22 (1.04, 1.43)^*^ | 1.07 (0.89, 1.29) |
| Missing | 0.92 (0.80, 1.06) | 1.04 (0.85, 1.28) | 0.89 (0.79, 1.00)^*^ | 1.15 (0.69, 1.90) | 0.67 (0.48, 0.94)^*^ | 1.32 (0.93, 1.88) | 1.05 (0.73, 1.51) | 1.00 (0.67, 1.47) |
| Often feeling lonely |  |  |  |  |  |  |  |  |
| No | Ref. | Ref. | Ref. | Ref. | Ref. | Ref. | Ref. | Ref. |
| Yes | 1.34 (1.28, 1.41)^***^ | 1.31 (1.20, 1.43)^***^ | 1.21 (1.15, 1.26)^***^ | 1.35 (1.12, 1.63)^**^ | 1.15 (1.03, 1.28)^**^ | 0.96 (0.81, 1.14) | 0.83 (0.71, 0.97)^*^ | 0.96 (0.81, 1.13) |
| Missing | 1.20 (1.00, 1.44) | 1.16 (0.84, 1.62) | 1.12 (0.94, 1.33) | 1.37 (0.68, 2.77) | 1.17 (0.76, 1.78) | 0.71 (0.35, 1.43) | 0.57 (0.31, 1.08) | 1.38 (0.71, 2.69) |
| Friend/family visits |  |  |  |  |  |  |  |  |
| Never or almost never | Ref. | Ref. | Ref. | Ref. | Ref. | Ref. | Ref. | Ref. |
| Once every 3 months | 0.74 (0.63, 0.87)^***^ | 0.71 (0.55, 0.93)^*^ | 0.74 (0.65, 0.84)^***^ | 1.08 (0.61, 1.92) | 0.78 (0.58, 1.04) | 1.30 (0.76, 2.22) | 0.91 (0.59, 1.41) | 1.79 (1.06, 3.01)^*^ |
| Monthly | 0.69 (0.59, 0.80)^***^ | 0.63 (0.49, 0.81)^***^ | 0.63 (0.56, 0.70)^***^ | 0.89 (0.51, 1.53) | 0.79 (0.61, 1.03) | 1.32 (0.79, 2.19) | 1.11 (0.74, 1.65) | 1.62 (0.98, 2.68) |
| Weekly | 0.64 (0.55, 0.74)^***^ | 0.57 (0.45, 0.72)^***^ | 0.61 (0.54, 0.68)^***^ | 0.86 (0.52, 1.44) | 0.75 (0.59, 0.97)^*^ | 1.12 (0.69, 1.81) | 0.98 (0.67, 1.43) | 1.74 (1.08, 2.80)^*^ |
| 2-4 times a week | 0.62 (0.54, 0.72)^***^ | 0.63 (0.50, 0.80)^***^ | 0.61 (0.55, 0.69)^***^ | 0.87 (0.52, 1.45) | 0.65 (0.51, 0.83)^***^ | 1.15 (0.71, 1.85) | 0.99 (0.68, 1.44) | 1.61 (1.01, 2.59)^*^ |
| Daily | 0.68 (0.59, 0.79)^***^ | 0.64 (0.51, 0.82)^***^ | 0.62 (0.55, 0.69)^***^ | 0.80 (0.47, 1.37) | 0.65 (0.50, 0.84)^**^ | 1.24 (0.76, 2.02) | 1.00 (0.68, 1.47) | 1.39 (0.85, 2.26) |
| Missing | 0.57 (0.37, 0.89)^*^ | 0.64 (0.33, 1.24) | 0.84 (0.61, 1.14) | 0.54 (0.07, 4.08) | 0.36 (0.11, 1.16) | 2.33 (0.78, 6.95) | 2.71 (1.04, 7.07)^*^ | 0.23 (0.03, 1.75) |
| Weekly social activity |  |  |  |  |  |  |  |  |
| No | Ref. | Ref. | Ref. | Ref. | Ref. | Ref. | Ref. | Ref. |
| Yes | 0.99 (0.95, 1.04) | 0.85 (0.78, 0.91)^***^ | 0.84 (0.80, 0.87)^***^ | 0.87 (0.73, 1.04) | 0.79 (0.71, 0.87)^***^ | 0.96 (0.83, 1.11) | 0.91 (0.80, 1.03) | 0.99 (0.85, 1.15) |
| Missing | 0.81 (0.49, 1.35) | 1.22 (0.63, 2.36) | 1.18 (0.81, 1.72) | 3.34 (1.06, 10.49)^*^ | 1.30 (0.42, 4.05) | 0.79 (0.20, 3.19) | 0.96 (0.31, 3.01) | 2.15 (0.79, 5.86) |
| Living alone |  |  |  |  |  |  |  |  |
| No | Ref. | Ref. | Ref. | Ref. | Ref. | Ref. | Ref. | Ref. |
| Yes | 1.43 (1.36, 1.49)^***^ | 1.16 (1.06, 1.26)^***^ | 1.38 (1.32, 1.44)^***^ | 1.37 (1.15, 1.63)^***^ | 1.26 (1.14, 1.39)^***^ | 1.08 (0.92, 1.27) | 0.90 (0.78, 1.04) | 0.85 (0.73, 1.00) |
| Missing | 1.69 (1.43, 2.00)^***^ | 1.30 (0.96, 1.78) | 1.64 (1.41, 1.90)^***^ | 1.36 (0.75, 2.49) | 1.62 (1.20, 2.20)^**^ | 0.62 (0.31, 1.26) | 0.78 (0.46, 1.33) | 0.77 (0.38, 1.56) |

^a^Model was adjusted for age, sex, education, multimorbidity, hearing, vision, handgrip strength and heavy drinking.

^b^Model was adjusted for age, sex, education, multimorbidity, hearing, vision, handgrip strength, heavy drinking, and presence of APOE4-allele

HR=hazard ratio; CI=confidence interval; PA = physical activity; MET = metabolic task equivalent.

^*^P<0.05; ^**^P<0.01 ; ^***^P<0.001

**S-Table 7.** Physical activity and social isolation in non-working individuals by multistate transitions, excluding falls and dementia within ten years of baseline

| Leisure physical activity by transition in non-working individuals (n=116 232) | | | | | | | | |
| --- | --- | --- | --- | --- | --- | --- | --- | --- |
|  | **Healthy to fall (n=4491)^a^** | **Healthy to dementia (n=1848)^b^** | **Healthy to death (n=12 901)^a^** | **Fall to fall & dementia (n=190)^b^** | **Fall to death (n=697)^a^** | **Dementia to fall & dementia (n=481)^b^** | **Dementia to death (n=387)^b^** | **Fall & dementia to death (n=284)^b^** |
| Walking |  |  |  |  |  |  |  |  |
| <600 MET-min/week | Ref. | Ref. | Ref. | Ref. | Ref. | Ref. | Ref. | Ref. |
| 600-1200 MET-min/week | 0.98 (0.91, 1.06) | 0.97 (0.86, 1.09) | 0.86 (0.82, 0.90)^***^ | 0.65 (0.43, 0.96)^*^ | 1.02 (0.85, 1.23) | 1.05 (0.84, 1.32) | 1.17 (0.95, 1.46) | 1.01 (0.75, 1.37) |
| >1200 MET-min/week | 0.97 (0.90, 1.03) | 1.01 (0.91, 1.12) | 0.84 (0.81, 0.88)^***^ | 0.99 (0.71, 1.37) | 0.94 (0.78, 1.12) | 0.83 (0.67, 1.03) | 0.94 (0.76, 1.15) | 0.78 (0.59, 1.04) |
| Moderate PA |  |  |  |  |  |  |  |  |
| <600 MET-min/week | Ref. | Ref. | Ref. | Ref. | Ref. | Ref. | Ref. | Ref. |
| 600-1200 MET-min/week | 0.91 (0.84, 0.98)^*^ | 0.90 (0.80, 1.03) | 0.83 (0.80, 0.88)^***^ | 0.95 (0.64, 1.41) | 0.84 (0.68, 1.04) | 0.91 (0.71, 1.16) | 0.96 (0.75, 1.23) | 0.68 (0.48, 0.96)^*^ |
| >1200 MET-min/week | 0.90 (0.84, 0.96)^**^ | 0.94 (0.85, 1.04) | 0.83 (0.80, 0.87)^***^ | 0.98 (0.71, 1.37) | 0.99 (0.83, 1.17) | 0.82 (0.67, 1.01) | 1.03 (0.85, 1.25) | 0.76 (0.58, 1.01) |
| Vigorous PA |  |  |  |  |  |  |  |  |
| <600 MET-min/week | Ref. | Ref. | Ref. | Ref. | Ref. | Ref. | Ref. | Ref. |
| 600-1200 MET-min/week | 0.91 (0.83, 0.99)^*^ | 0.93 (0.81, 1.07) | 0.79 (0.75, 0.83)^***^ | 0.89 (0.56, 1.39) | 0.92 (0.72, 1.17) | 0.71 (0.52, 0.96)^*^ | 0.88 (0.67, 1.16) | 0.75 (0.50, 1.13) |
| >1200 MET-min/week | 0.91 (0.84, 0.99)^*^ | 1.02 (0.90, 1.15) | 0.77 (0.73, 0.81)^***^ | 0.84 (0.55, 1.29) | 0.99 (0.80, 1.22) | 0.87 (0.68, 1.12) | 0.94 (0.75, 1.19) | 1.08 (0.79, 1.49) |
| Moderate-to-vigorous PA |  |  |  |  |  |  |  |  |
| <600 MET-min/week | Ref. | Ref. | Ref. | Ref. | Ref. | Ref. | Ref. | Ref. |
| 600-1200 MET-min/week | 0.86 (0.79, 0.93)^***^ | 0.89 (0.78, 1.02) | 0.79 (0.75, 0.83)^***^ | 1.01 (0.67, 1.50) | 0.71 (0.56, 0.89)^**^ | 0.80 (0.61, 1.04) | 0.94 (0.74, 1.21) | 0.95 (0.67, 1.33) |
| >1200 MET-min/week | 0.86 (0.81, 0.92)^***^ | 0.88 (0.80, 0.98)^*^ | 0.75 (0.72, 0.78)^***^ | 0.79 (0.58, 1.09) | 0.90 (0.77, 1.06) | 0.71 (0.58, 0.87)^***^ | 0.85 (0.70, 1.03) | 0.79 (0.61, 1.03) |

| Social isolation by transition in non-working individuals (n=116 232) | | | | | | | | |
| --- | --- | --- | --- | --- | --- | --- | --- | --- |
|  | **Healthy to fall (n=4491)^a^** | **Healthy to dementia (n=1848)^b^** | **Healthy to death (n=12 901)^a^** | **Fall to fall & dementia (n=190)^b^** | **Fall to death (n=697)^a^** | **Dementia to fall & dementia (n=481)^b^** | **Dementia to death (n=387)^b^** | **Fall & dementia to death (n=284)^b^** |
|  | HRs (95% CI) | HRs (95% CI) | HRs (95% CI) | HRs (95% CI) | HRs (95% CI) | HRs (95% CI) | HRs (95% CI) | HRs (95% CI) |
| Ability to confide |  |  |  |  |  |  |  |  |
| Never or almost never | Ref. | Ref. | Ref. | Ref. | Ref. | Ref. | Ref. | Ref. |
| Once every few months | 0.92 (0.79, 1.06) | 1.07 (0.87, 1.31) | 0.91 (0.83, 0.98)^*^ | 1.74 (0.97, 3.11) | 0.59 (0.39, 0.90)^*^ | 1.59 (1.09, 2.31)^*^ | 1.25 (0.84, 1.88) | 0.77 (0.48, 1.23) |
| About once a month | 0.96 (0.83, 1.11) | 0.99 (0.79, 1.24) | 0.86 (0.79, 0.94)^***^ | 1.48 (0.78, 2.78) | 0.76 (0.52, 1.13) | 1.02 (0.66, 1.59) | 1.26 (0.83, 1.91) | 0.96 (0.56, 1.65) |
| About once a week | 0.92 (0.82, 1.04) | 0.99 (0.83, 1.18) | 0.90 (0.84, 0.97)^**^ | 0.92 (0.49, 1.74) | 1.01 (0.76, 1.36) | 0.99 (0.70, 1.40) | 0.95 (0.66, 1.36) | 0.83 (0.51, 1.34) |
| 2-4 times a week | 1.04 (0.92, 1.18) | 0.84 (0.69, 1.03) | 0.86 (0.80, 0.93)^***^ | 1.46 (0.83, 2.57) | 0.91 (0.66, 1.26) | 1.06 (0.71, 1.59) | 1.03 (0.68, 1.57) | 0.68 (0.39, 1.18) |
| Daily or almost daily | 0.92 (0.84, 1.00)^*^ | 0.83 (0.73, 0.94)^**^ | 0.83 (0.79, 0.87)^***^ | 1.02 (0.67, 1.54) | 1.01 (0.82, 1.24) | 0.99 (0.76, 1.28) | 1.34 (1.05, 1.72)^*^ | 0.83 (0.60, 1.15) |
| Missing | 0.85 (0.69, 1.06) | 1.19 (0.91, 1.57) | 0.89 (0.79, 1.00) | 1.31 (0.54, 3.14) | 0.52 (0.27, 1.00)^*^ | 1.57 (0.95, 2.61) | 1.56 (0.93, 2.61) | 1.03 (0.53, 1.99) |
| Often feeling lonely |  |  |  |  |  |  |  |  |
| No | Ref. | Ref. | Ref. | Ref. | Ref. | Ref. | Ref. | Ref. |
| Yes | 1.30 (1.21, 1.40)^***^ | 1.20 (1.06, 1.36)^**^ | 1.22 (1.17, 1.28)^***^ | 1.34 (0.95, 1.91) | 1.09 (0.90, 1.32) | 1.07 (0.84, 1.36) | 0.71 (0.55, 0.93)^*^ | 1.10 (0.81, 1.51) |
| Missing | 1.32 (1.01, 1.73)^*^ | 1.08 (0.68, 1.72) | 1.12 (0.94, 1.33) | 1.22 (0.39, 3.87) | 0.99 (0.51, 1.93) | 0.34 (0.08, 1.36) | 0.68 (0.25, 1.82) | 0.00 (0.00, Inf) |
| Friend/family visits |  |  |  |  |  |  |  |  |
| Never or almost never | Ref. | Ref. | Ref. | Ref. | Ref. | Ref. | Ref. | Ref. |
| Once every 3 months | 0.81 (0.63, 1.06) | 0.92 (0.61, 1.40) | 0.73 (0.64, 0.83)^***^ | 1.03 (0.21, 4.98) | 0.57 (0.33, 0.97)^*^ | 2.00 (0.76, 5.25) | 0.91 (0.44, 1.89) | 0.78 (0.25, 2.42) |
| Monthly | 0.71 (0.56, 0.91)^**^ | 0.83 (0.56, 1.23) | 0.61 (0.54, 0.69)^***^ | 1.63 (0.38, 7.01) | 0.74 (0.45, 1.20) | 2.13 (0.84, 5.42) | 0.99 (0.50, 1.96) | 0.96 (0.33, 2.78) |
| Weekly | 0.71 (0.56, 0.90)^**^ | 0.76 (0.52, 1.10) | 0.59 (0.53, 0.66)^***^ | 1.53 (0.37, 6.31) | 0.62 (0.40, 0.98)^*^ | 1.70 (0.68, 4.23) | 0.87 (0.46, 1.67) | 0.97 (0.35, 2.69) |
| 2-4 times a week | 0.66 (0.53, 0.84)^***^ | 0.79 (0.54, 1.15) | 0.60 (0.54, 0.67)^***^ | 1.92 (0.47, 7.88) | 0.63 (0.40, 1.00)^*^ | 1.71 (0.69, 4.24) | 0.84 (0.44, 1.61) | 0.92 (0.33, 2.55) |
| Daily | 0.73 (0.57, 0.92)^**^ | 0.82 (0.56, 1.20) | 0.61 (0.54, 0.68)^***^ | 0.95 (0.22, 4.13) | 0.60 (0.37, 0.97)^*^ | 1.47 (0.58, 3.69) | 0.96 (0.50, 1.86) | 0.66 (0.23, 1.91) |
| Missing | 0.60 (0.30, 1.21) | 0.64 (0.23, 1.83) | 0.81 (0.60, 1.11) | 0.00 (0.00, Inf) | 0.31 (0.04, 2.35) | 443.35 (33.92, 5794.70)^***^ | 688.82 (102.16, 4644.29)^***^ | 0.00 (0.00, Inf) |
| Weekly social activity |  |  |  |  |  |  |  |  |
| No | Ref. | Ref. | Ref. | Ref. | Ref. | Ref. | Ref. | Ref. |
| Yes | 0.98 (0.92, 1.05) | 0.87 (0.78, 0.96)^**^ | 0.83 (0.80, 0.87)^***^ | 0.73 (0.53, 1.00) | 0.73 (0.62, 0.86)^***^ | 0.93 (0.76, 1.14) | 0.79 (0.65, 0.95)^*^ | 0.94 (0.72, 1.24) |
| Missing | 1.05 (0.52, 2.10) | 1.11 (0.41, 2.97) | 1.17 (0.81, 1.70) | 0.00 (0.00, Inf) | 2.63 (0.84, 8.29) | 0.00 (0.00, Inf) | 0.85 (0.12, 6.17) | NA |
| Living alone |  |  |  |  |  |  |  |  |
| No | Ref. | Ref. | Ref. | Ref. | Ref. | Ref. | Ref. | Ref. |
| Yes | 1.37 (1.28, 1.47)^***^ | 1.17 (1.05, 1.32)^**^ | 1.39 (1.34, 1.45)^***^ | 1.10 (0.79, 1.54) | 1.12 (0.94, 1.33) | 0.93 (0.74, 1.17) | 0.90 (0.72, 1.13) | 0.81 (0.60, 1.09) |
| Missing | 1.87 (1.46, 2.39)^***^ | 1.45 (0.96, 2.19) | 1.67 (1.44, 1.93)^***^ | 1.02 (0.32, 3.25) | 1.27 (0.74, 2.17) | 0.69 (0.25, 1.85) | 0.85 (0.38, 1.92) | 0.87 (0.21, 3.54) |

^a^Model was adjusted for age, sex, education, multimorbidity, hearing, vision, handgrip strength and heavy drinking.

^b^Model was adjusted for age, sex, education, multimorbidity, hearing, vision, handgrip strength, heavy drinking, and presence of APOE4-allele

HR=hazard ratio; CI=confidence interval; PA = physical activity; MET = metabolic task equivalent.

^*^P<0.05; ^**^P<0.01 ; ^***^P<0.001

**S-Table 8.** Activity profile membership by transitions in full and stratified samples (working vs. non-working), with additional covariates included

| Activity profile membership by transition in full sample (n=288 287) | | | | | | | | |
| --- | --- | --- | --- | --- | --- | --- | --- | --- |
|  | **Healthy to fall (n=19 753)^a^** | **Healthy to dementia (n=4407)^b^** | **Healthy to death (n=19 723)^a^** | **Fall to fall & dementia (n=903)^b^** | **Fall to death (n=3141)^a^** | **Dementia to fall & dementia (n=1146)^b^** | **Dementia to death (n=1608)^b^** | **Fall & dementia to death (n=1111)^b^** |
|  | HRs (95% CI) | HRs (95% CI) | HRs (95% CI) | HRs (95% CI) | HRs (95% CI) | HRs (95% CI) | HRs (95% CI) | HRs (95% CI) |
| Active, working profile | Ref. | Ref. | Ref. | Ref. | Ref. | Ref. | Ref. | Ref. |
| Non-active, working profile | 1.00 (0.96, 1.05) | 1.02 (0.89, 1.16) | 1.01 (0.96, 1.06) | 1.36 (0.96, 1.93) | 1.03 (0.90, 1.20) | 0.94 (0.70, 1.25) | 1.08 (0.86, 1.36) | 1.19 (0.85, 1.64) |
| Active, non-working profile | 1.09 (1.04, 1.14)^***^ | 1.39 (1.24, 1.55)^***^ | 1.14 (1.09, 1.20)^***^ | 1.87 (1.38, 2.52)^***^ | 1.22 (1.07, 1.39)^**^ | 1.02 (0.81, 1.29) | 1.05 (0.87, 1.27) | 1.09 (0.83, 1.42) |
| Non-active, non-working profile | 1.39 (1.32, 1.46)^***^ | 1.67 (1.48, 1.88)^***^ | 1.47 (1.40, 1.54)^***^ | 2.35 (1.73, 3.18)^***^ | 1.45 (1.27, 1.65)^***^ | 1.19 (0.93, 1.52) | 1.01 (0.83, 1.24) | 1.07 (0.81, 1.41) |
| Activity profile membership by transition in non-working profiles (n=125 481) | | | | | | | | |
|  | **Healthy to fall (n=12 073)^a^** | **Healthy to dementia (n=3515)^b^** | **Healthy to death (n=12 901)^a^** | **Fall to fall & dementia (n=771)^b^** | **Fall to death (n=2391)^a^** | **Dementia to fall & dementia (n=964)^b^** | **Dementia to death (n=1314)^b^** | **Fall & dementia to death (n=962)^b^** |
|  | HRs (95% CI) | HRs (95% CI) | HRs (95% CI) | HRs (95% CI) | HRs (95% CI) | HRs (95% CI) | HRs (95% CI) | HRs (95% CI) |
| Active, non-working profile | Ref. | Ref. | Ref. | Ref. | Ref. | Ref. | Ref. | Ref. |
| Non-active, non-working profile | 1.25 (1.21, 1.30)^***^ | 1.21 (1.12, 1.29)^***^ | 1.28 (1.23, 1.33)^***^ | 1.25 (1.07, 1.45)^**^ | 1.19 (1.09, 1.30)^***^ | 1.19 (1.04, 1.37)^*^ | 0.97 (0.86, 1.10) | 0.99 (0.86, 1.13) |
| Activity profile membership by transition in working profiles (n=162 806) | | | | | | | | |
|  | **Healthy to fall (n=7680)^a^** | **Healthy to dementia (n=892)^b^** | **Healthy to death (n=6822)^a^** | **Fall to fall & dementia (n=132)^b^** | **Fall to death (n=750)^a^** | **Dementia to fall & dementia (n=182)^b^** | **Dementia to death (n=294)^b^** | **Fall & dementia to death (n=149)^b^** |
|  | HRs (95% CI) | HRs (95% CI) | HRs (95% CI) | HRs (95% CI) | HRs (95% CI) | HRs (95% CI) | HRs (95% CI) | HRs (95% CI) |
| Active, working profile | Ref. | Ref. | Ref. | Ref. | Ref. | Ref. | Ref. | Ref. |
| Non-active, working profile | 0.99 (0.95, 1.04) | 1.03 (0.90, 1.17) | 0.99 (0.94, 1.04) | 1.40 (0.98, 2.00) | 1.04 (0.90, 1.21) | 1.00 (0.74, 1.35) | 1.13 (0.89, 1.43) | 1.15 (0.81, 1.64) |

^a^Model was adjusted for age, sex, education, Charlson Comorbidity Index, hearing, vision, handgrip strength, heavy drinking, smoking, body mass index, and systolic blood pressure.

^b^Model was adjusted for age, sex, education, Charlson Comorbidity Index, hearing, vision, handgrip strength, heavy drinking, smoking, body mass index, systolic blood pressure, and presence of APOE4-allele

HR=hazard ratio; CI=confidence interval.^*^P<0.05; ^**^P<0.01; ^***^P<0.001

**S-Table 9.** Physical activity and social isolation in non-working individuals by transitions, with additional covariates included

| Physical activity by transition in non-working individuals (n=125 481) | | | | | | | | |
| --- | --- | --- | --- | --- | --- | --- | --- | --- |
|  | **Healthy to fall (n=12 073)^a^** | **Healthy to dementia (n=3515)^b^** | **Healthy to death (n=12 901)^a^** | **Fall to fall & dementia (n=771)^b^** | **Fall to death (n=2391)^a^** | **Dementia to fall & dementia (n=964)^b^** | **Dementia to death (n=1314)^b^** | **Fall & dementia to death (n=962)^b^** |
| Walking |  |  |  |  |  |  |  |  |
| <600 MET-min/week | Ref. | Ref. | Ref. | Ref. | Ref. | Ref. | Ref. | Ref. |
| 600-1200 MET-min/week | 0.99 (0.94, 1.03) | 0.98 (0.90, 1.06) | 0.89 (0.85, 0.93)^***^ | 0.95 (0.79, 1.15) | 1.00 (0.90, 1.10) | 1.01 (0.86, 1.18) | 1.05 (0.91, 1.20) | 1.02 (0.87, 1.20) |
| >1200 MET-min/week | 0.99 (0.94, 1.03) | 0.96 (0.89, 1.04) | 0.87 (0.83, 0.91)^***^ | 0.93 (0.79, 1.10) | 0.87 (0.79, 0.96)^**^ | 0.87 (0.75, 1.02) | 1.00 (0.88, 1.15) | 0.90 (0.76, 1.05) |
| Moderate PA |  |  |  |  |  |  |  |  |
| <600 MET-min/week | Ref. | Ref. | Ref. | Ref. | Ref. | Ref. | Ref. | Ref. |
| 600-1200 MET-min/week | 0.89 (0.85, 0.94)^***^ | 0.91 (0.83, 1.00)^*^ | 0.87 (0.83, 0.91)^***^ | 0.85 (0.69, 1.04) | 0.83 (0.74, 0.94)^**^ | 0.95 (0.80, 1.13) | 0.98 (0.84, 1.14) | 1.00 (0.83, 1.20) |
| >1200 MET-min/week | 0.92 (0.88, 0.96)^***^ | 0.92 (0.86, 1.00)^*^ | 0.86 (0.83, 0.90)^***^ | 0.93 (0.79, 1.09) | 0.92 (0.84, 1.01) | 0.86 (0.74, 1.00)^*^ | 0.95 (0.84, 1.08) | 0.90 (0.78, 1.05) |
| Vigorous PA |  |  |  |  |  |  |  |  |
| <600 MET-min/week | Ref. | Ref. | Ref. | Ref. | Ref. | Ref. | Ref. | Ref. |
| 600-1200 MET-min/week | 0.92 (0.87, 0.97)^**^ | 0.96 (0.87, 1.06) | 0.83 (0.78, 0.88)^***^ | 0.79 (0.62, 1.01) | 0.88 (0.77, 1.01) | 0.67 (0.54, 0.83)^***^ | 0.96 (0.81, 1.13) | 0.90 (0.72, 1.13) |
| >1200 MET-min/week | 0.90 (0.86, 0.95)^***^ | 1.01 (0.92, 1.10) | 0.82 (0.78, 0.86)^***^ | 0.92 (0.74, 1.13) | 0.83 (0.73, 0.94)^**^ | 0.95 (0.80, 1.13) | 0.91 (0.78, 1.06) | 0.96 (0.81, 1.15) |
| Moderate-to-vigorous PA |  |  |  |  |  |  |  |  |
| <600 MET-min/week | Ref. | Ref. | Ref. | Ref. | Ref. | Ref. | Ref. | Ref. |
| 600-1200 MET-min/week | 0.87 (0.82, 0.91)^***^ | 0.90 (0.82, 0.99)^*^ | 0.83 (0.79, 0.88)^***^ | 0.95 (0.77, 1.16) | 0.80 (0.71, 0.90)^***^ | 0.82 (0.68, 0.99)^*^ | 0.97 (0.83, 1.14) | 1.14 (0.95, 1.38) |
| >1200 MET-min/week | 0.87 (0.83, 0.90)^***^ | 0.89 (0.82, 0.95)^**^ | 0.80 (0.77, 0.83)^***^ | 0.85 (0.72, 1.00)^*^ | 0.86 (0.78, 0.94)^***^ | 0.81 (0.70, 0.94)^**^ | 0.88 (0.78, 1.00)^*^ | 0.91 (0.78, 1.05) |

| Social isolation by transition in non-working individuals (n=125 481) | | | | | | | | |
| --- | --- | --- | --- | --- | --- | --- | --- | --- |
|  | **Healthy to fall (n=12 073)^a^** | **Healthy to dementia (n=3515)^b^** | **Healthy to death (n=12 901)^a^** | **Fall to fall & dementia (n=771)^b^** | **Fall to death (n=2391)^a^** | **Dementia to fall & dementia (n=964)^b^** | **Dementia to death (n=1314)^b^** | **Fall & dementia to death (n=962)^b^** |
|  | HRs (95% CI) | HRs (95% CI) | HRs (95% CI) | HRs (95% CI) | HRs (95% CI) | HRs (95% CI) | HRs (95% CI) | HRs (95% CI) |
| Ability to confide |  |  |  |  |  |  |  |  |
| Never or almost never | Ref. | Ref. | Ref. | Ref. | Ref. | Ref. | Ref. | Ref. |
| Once every few months | 0.99 (0.91, 1.08) | 0.98 (0.84, 1.14) | 0.93 (0.86, 1.01) | 0.99 (0.72, 1.37) | 0.88 (0.73, 1.06) | 1.26 (0.97, 1.65) | 1.07 (0.82, 1.39) | 0.85 (0.64, 1.13) |
| About once a month | 0.99 (0.91, 1.08) | 1.08 (0.92, 1.26) | 0.90 (0.82, 0.98)^*^ | 1.17 (0.84, 1.62) | 1.17 (0.97, 1.41) | 0.92 (0.69, 1.24) | 1.19 (0.93, 1.53) | 0.84 (0.63, 1.13) |
| About once a week | 0.93 (0.87, 1.00) | 0.96 (0.85, 1.09) | 0.93 (0.87, 0.99)^*^ | 1.04 (0.79, 1.36) | 1.05 (0.90, 1.22) | 0.80 (0.63, 1.02) | 0.83 (0.66, 1.03) | 0.82 (0.64, 1.04) |
| 2-4 times a week | 0.99 (0.92, 1.07) | 0.85 (0.74, 0.99)^*^ | 0.89 (0.83, 0.96)^**^ | 1.27 (0.96, 1.67) | 0.96 (0.81, 1.14) | 0.87 (0.67, 1.14) | 0.92 (0.72, 1.17) | 0.96 (0.73, 1.25) |
| Daily or almost daily | 0.87 (0.83, 0.92)^***^ | 0.77 (0.71, 0.85)^***^ | 0.87 (0.83, 0.91)^***^ | 0.88 (0.72, 1.07) | 0.98 (0.88, 1.09) | 0.83 (0.69, 0.99)^*^ | 1.17 (1.01, 1.37)^*^ | 1.08 (0.91, 1.28) |
| Missing | 0.95 (0.84, 1.07) | 1.06 (0.87, 1.30) | 0.93 (0.82, 1.04) | 1.09 (0.71, 1.69) | 0.70 (0.52, 0.93)^*^ | 1.27 (0.89, 1.80) | 0.92 (0.64, 1.32) | 1.01 (0.70, 1.46) |
| Often feeling lonely |  |  |  |  |  |  |  |  |
| No | Ref. | Ref. | Ref. | Ref. | Ref. | Ref. | Ref. | Ref. |
| Yes | 1.30 (1.24, 1.36)^***^ | 1.30 (1.19, 1.42)^***^ | 1.12 (1.07, 1.17)^***^ | 1.46 (1.23, 1.72)^***^ | 1.06 (0.96, 1.17) | 0.95 (0.81, 1.13) | 0.87 (0.74, 1.01) | 0.94 (0.81, 1.11) |
| Missing | 1.11 (0.93, 1.33) | 1.09 (0.78, 1.52) | 1.09 (0.92, 1.30) | 1.57 (0.84, 2.94) | 1.26 (0.86, 1.84) | 0.71 (0.33, 1.50) | 0.72 (0.39, 1.31) | 1.21 (0.62, 2.36) |
| Friend/family visits |  |  |  |  |  |  |  |  |
| Never or almost never | Ref. | Ref. | Ref. | Ref. | Ref. | Ref. | Ref. | Ref. |
| Once every 3 months | 0.74 (0.64, 0.85)^***^ | 0.77 (0.59, 1.01) | 0.80 (0.70, 0.91)^***^ | 1.00 (0.63, 1.60) | 0.87 (0.67, 1.14) | 1.19 (0.70, 2.04) | 0.84 (0.55, 1.28) | 2.01 (1.24, 3.26)^**^ |
| Monthly | 0.69 (0.60, 0.79)^***^ | 0.67 (0.52, 0.86)^**^ | 0.69 (0.61, 0.78)^***^ | 0.77 (0.49, 1.20) | 1.01 (0.79, 1.29) | 1.30 (0.78, 2.16) | 1.09 (0.74, 1.62) | 2.00 (1.25, 3.21)^**^ |
| Weekly | 0.65 (0.57, 0.73)^***^ | 0.60 (0.48, 0.76)^***^ | 0.67 (0.60, 0.75)^***^ | 0.73 (0.48, 1.09) | 0.89 (0.71, 1.12) | 1.15 (0.71, 1.87) | 0.99 (0.68, 1.43) | 1.88 (1.21, 2.92)^**^ |
| 2-4 times a week | 0.63 (0.56, 0.72)^***^ | 0.67 (0.53, 0.85)^***^ | 0.68 (0.61, 0.76)^***^ | 0.78 (0.52, 1.16) | 0.78 (0.62, 0.98)^*^ | 1.15 (0.71, 1.86) | 1.01 (0.70, 1.46) | 1.76 (1.13, 2.73)^*^ |
| Daily | 0.68 (0.60, 0.78)^***^ | 0.67 (0.53, 0.86)^**^ | 0.66 (0.59, 0.74)^***^ | 0.68 (0.44, 1.05) | 0.78 (0.61, 0.99)^*^ | 1.29 (0.79, 2.10) | 1.02 (0.70, 1.49) | 1.51 (0.95, 2.38) |
| Missing | 0.56 (0.38, 0.84)^**^ | 0.64 (0.33, 1.24) | 0.86 (0.63, 1.17) | 0.77 (0.18, 3.24) | 0.43 (0.16, 1.18) | 2.54 (0.84, 7.71) | 3.15 (1.20, 8.25)^*^ | 0.62 (0.14, 2.68) |
| Weekly social activity |  |  |  |  |  |  |  |  |
| No | Ref. | Ref. | Ref. | Ref. | Ref. | Ref. | Ref. | Ref. |
| Yes | 1.03 (0.99, 1.07) | 0.86 (0.80, 0.93)^***^ | 0.90 (0.86, 0.93)^***^ | 0.92 (0.78, 1.09) | 0.82 (0.75, 0.90)^***^ | 0.96 (0.83, 1.11) | 0.90 (0.80, 1.02) | 1.09 (0.94, 1.26) |
| Missing | 1.01 (0.66, 1.54) | 1.31 (0.70, 2.45) | 1.22 (0.84, 1.78) | 1.72 (0.54, 5.51) | 0.74 (0.30, 1.80) | 0.42 (0.10, 1.72) | 0.39 (0.12, 1.22) | 2.79 (1.02, 7.63)^*^ |
| Living alone |  |  |  |  |  |  |  |  |
| No | Ref. | Ref. | Ref. | Ref. | Ref. | Ref. | Ref. | Ref. |
| Yes | 1.40 (1.34, 1.46)^***^ | 1.14 (1.05, 1.24)^**^ | 1.27 (1.22, 1.33)^***^ | 1.29 (1.10, 1.51)^**^ | 1.15 (1.05, 1.26)^**^ | 1.08 (0.92, 1.26) | 0.90 (0.78, 1.04) | 0.83 (0.72, 0.97)^*^ |
| Missing | 1.68 (1.45, 1.96)^***^ | 1.42 (1.07, 1.90)^*^ | 1.45 (1.25, 1.68)^***^ | 1.79 (1.14, 2.81)^*^ | 1.34 (1.02, 1.76)^*^ | 0.62 (0.32, 1.20) | 0.84 (0.51, 1.36) | 0.68 (0.39, 1.19) |

^a^Model was adjusted for age, sex, education, multimorbidity, hearing, vision, handgrip strength, heavy drinking, smoking, body mass index, and systolic blood pressure.

^b^Model was adjusted for age, sex, education, multimorbidity, hearing, vision, handgrip strength, heavy drinking, smoking, body mass index, systolic blood pressure, and presence of APOE4-allele

HR=hazard ratio; CI=confidence interval; PA = physical activity; MET = metabolic task equivalent

^*^P<0.05; ^**^P<0.01; ^***^P<0.001

**S-Table 10.** Activity profile membership and transitions in the full sample, non-working sub-group, with non-retired/non-working individuals excluded

| Activity profile membership by transition in full sample (n=264 588) | | | | | | | | |
| --- | --- | --- | --- | --- | --- | --- | --- | --- |
|  | **Healthy to fall (n=17 811)^a^** | **Healthy to dementia (n=4122)^b^** | **Healthy to death (n=17 614)^a^** | **Fall to fall & dementia (n=819)^b^** | **Fall to death (n=2735)^a^** | **Dementia to fall & dementia (n=1085)^b^** | **Dementia to death (n=1517)^b^** | **Fall & dementia to death (n=1045)^b^** |
|  | HRs (95% CI) | HRs (95% CI) | HRs (95% CI) | HRs (95% CI) | HRs (95% CI) | HRs (95% CI) | HRs (95% CI) | HRs (95% CI) |
| Active, working profile | Ref. | Ref. | Ref. | Ref. | Ref. | Ref. | Ref. | Ref. |
| Non-active, working profile | 1.00 (0.96, 1.05) | 1.01 (0.89, 1.16) | 1.02 (0.97, 1.07) | 1.37 (0.97, 1.95) | 1.05 (0.91, 1.22) | 0.93 (0.69, 1.24) | 1.06 (0.84, 1.34) | 1.13 (0.82, 1.57) |
| Active, non-working profile | 1.01 (0.96, 1.06) | 1.28 (1.14, 1.44)^***^ | 1.01 (0.96, 1.06) | 1.69 (1.24, 2.30)^***^ | 1.11 (0.97, 1.27) | 1.07 (0.84, 1.36) | 1.09 (0.90, 1.33) | 1.20 (0.91, 1.58) |
| Non-active, non-working profile | 1.27 (1.21, 1.34)^***^ | 1.53 (1.35, 1.73)^***^ | 1.37 (1.30, 1.45)^***^ | 2.05 (1.50, 2.81)^***^ | 1.40 (1.21, 1.61)^***^ | 1.29 (1.00, 1.66)^*^ | 1.03 (0.83, 1.27) | 1.18 (0.89, 1.56) |
| Activity profile membership by transition in non-working profiles (n=101 782) | | | | | | | | |
|  | **Healthy to fall (n=10 131)^a^** | **Healthy to dementia (n=3230)^b^** | **Healthy to death (n=10 792)^a^** | **Fall to fall & dementia (n=687)^b^** | **Fall to death (n=1985)^a^** | **Dementia to fall & dementia (n=903)^b^** | **Dementia to death (n=1223)^b^** | **Fall & dementia to death (n=896)^b^** |
|  | HRs (95% CI) | HRs (95% CI) | HRs (95% CI) | HRs (95% CI) | HRs (95% CI) | HRs (95% CI) | HRs (95% CI) | HRs (95% CI) |
| Active, non-working profile | Ref. | Ref. | Ref. | Ref. | Ref. | Ref. | Ref. | Ref. |
| Non-active, non-working profile | 1.25 (1.20, 1.31)^***^ | 1.20 (1.11, 1.29)^***^ | 1.37 (1.31, 1.42)^***^ | 1.21 (1.04, 1.42)^*^ | 1.27 (1.16, 1.39)^***^ | 1.22 (1.06, 1.41)^**^ | 0.94 (0.83, 1.07) | 0.99 (0.86, 1.13) |

^a^Model was adjusted for age, sex, education, Charlson Comorbidity Index, hearing, vision, handgrip strength and heavy drinking.

^b^Model was adjusted for age, sex, education, Charlson Comorbidity Index, hearing, vision, handgrip strength, heavy drinking, and presence of APOE4-allele

HR=hazard ratio; CI=confidence interval.

^*^P<0.05; ^**^P<0.01; ^***^P<0.001

**S-Table 11.** Activity profile components and transitions in the non-working sub-group, with non-retired/non-working individuals excluded

| **Leisure physical activity by transition in non-working individuals (n=101 782)** | | | | | | | | | |
| --- | --- | --- | --- | --- | --- | --- | --- | --- | --- |
|  | **Healthy to fall (n=10 131)^a^** | **Healthy to dementia (n=3230)^b^** | **Healthy to death (n=10 792)^a^** | **Fall to fall & dementia (n=687)^b^** | **Fall to death (n=1985)^a^** | **Dementia to fall & dementia (n=903)^b^** | **Dementia to death (n=1223)^b^** | **Fall & dementia to death (n=896)^b^** |  |
| Walking |  |  |  |  |  |  |  |  |  |
| <600 MET-min/week | Ref. | Ref. | Ref. | Ref. | Ref. | Ref. | Ref. | Ref. |  |
| 600-1200 MET-min/week | 0.99 (0.94, 1.04) | 0.98 (0.90, 1.07) | 0.87 (0.83, 0.92)^***^ | 0.93 (0.77, 1.13) | 0.97 (0.87, 1.08) | 1.00 (0.85, 1.18) | 1.04 (0.90, 1.19) | 1.01 (0.86, 1.19) |  |
| >1200 MET-min/week | 0.98 (0.93, 1.02) | 0.98 (0.90, 1.06) | 0.85 (0.81, 0.89)^***^ | 0.92 (0.77, 1.10) | 0.84 (0.75, 0.93)^**^ | 0.86 (0.73, 1.00) | 0.97 (0.85, 1.11) | 0.96 (0.82, 1.13) |  |
| Moderate PA |  |  |  |  |  |  |  |  |  |
| <600 MET-min/week | Ref. | Ref. | Ref. | Ref. | Ref. | Ref. | Ref. | Ref. |  |
| 600-1200 MET-min/week | 0.88 (0.84, 0.93)^***^ | 0.92 (0.84, 1.01) | 0.86 (0.82, 0.91)^***^ | 0.84 (0.68, 1.03) | 0.82 (0.72, 0.93)^**^ | 0.95 (0.79, 1.14) | 0.98 (0.84, 1.14) | 1.03 (0.85, 1.24) |  |
| >1200 MET-min/week | 0.91 (0.87, 0.95)^***^ | 0.93 (0.86, 1.01) | 0.84 (0.81, 0.88)^***^ | 0.92 (0.78, 1.09) | 0.90 (0.82, 1.00)^*^ | 0.87 (0.75, 1.01) | 0.96 (0.85, 1.09) | 0.90 (0.78, 1.05) |  |
| Vigorous PA |  |  |  |  |  |  |  |  |  |
| <600 MET-min/week | Ref. | Ref. | Ref. | Ref. | Ref. | Ref. | Ref. | Ref. |  |
| 600-1200 MET-min/week | 0.93 (0.88, 0.99)^*^ | 0.97 (0.87, 1.07) | 0.81 (0.76, 0.86)^***^ | 0.82 (0.64, 1.05) | 0.89 (0.77, 1.02) | 0.66 (0.53, 0.82)^***^ | 0.94 (0.79, 1.11) | 0.89 (0.71, 1.12) |  |
| >1200 MET-min/week | 0.89 (0.84, 0.94)^***^ | 1.01 (0.92, 1.11) | 0.79 (0.74, 0.83)^***^ | 0.92 (0.74, 1.14) | 0.84 (0.74, 0.96)^*^ | 0.94 (0.79, 1.12) | 0.91 (0.78, 1.06) | 0.96 (0.80, 1.15) |  |
| Moderate-to-vigorous PA |  |  |  |  |  |  |  |  |  |
| <600 MET-min/week | Ref. | Ref. | Ref. | Ref. | Ref. | Ref. | Ref. | Ref. |  |
| 600-1200 MET-min/week | 0.87 (0.82, 0.92)^***^ | 0.89 (0.81, 0.99)^*^ | 0.81 (0.77, 0.86)^***^ | 0.96 (0.77, 1.18) | 0.78 (0.68, 0.89)^***^ | 0.78 (0.64, 0.95)^*^ | 0.98 (0.83, 1.16) | 1.14 (0.94, 1.38) |  |
| >1200 MET-min/week | 0.86 (0.82, 0.90)^***^ | 0.90 (0.83, 0.97)^**^ | 0.77 (0.74, 0.80)^***^ | 0.85 (0.72, 1.01) | 0.83 (0.75, 0.91)^***^ | 0.79 (0.69, 0.92)^**^ | 0.88 (0.77, 1.00)^*^ | 0.92 (0.80, 1.07) |  |
|  | **Social isolation by transition in non-working individuals (n=101 782)** | | | | | | | | |
|  | **Healthy to fall (n=10 131)^a^** | **Healthy to dementia (n=3230)^b^** | **Healthy to death (n=10 792)^a^** | **Fall to fall & dementia (n=687)^b^** | **Fall to death (n=1985)^a^** | **Dementia to fall & dementia (n=903)^b^** | **Dementia to death (n=1223)^b^** | **Fall & dementia to death (n=896)^b^** |  |
|  | **HRs (95% CI)** | **HRs (95% CI)** | **HRs (95% CI)** | **HRs (95% CI)** | **HRs (95% CI)** | **HRs (95% CI)** | **HRs (95% CI)** | **HRs (95% CI)** |  |
| Ability to confide |  |  |  |  |  |  |  |  |  |
| Never or almost never | Ref. | Ref. | Ref. | Ref. | Ref. | Ref. | Ref. | Ref. |  |
| Once every few months | 0.98 (0.90, 1.08) | 0.98 (0.84, 1.15) | 0.92 (0.84, 1.01) | 1.07 (0.76, 1.51) | 0.87 (0.71, 1.07) | 1.19 (0.90, 1.56) | 1.09 (0.83, 1.43) | 0.92 (0.69, 1.22) |  |
| About once a month | 0.97 (0.88, 1.06) | 1.09 (0.92, 1.28) | 0.84 (0.76, 0.93)^***^ | 1.22 (0.86, 1.72) | 1.09 (0.89, 1.35) | 0.87 (0.65, 1.18) | 1.23 (0.95, 1.59) | 0.79 (0.58, 1.07) |  |
| About once a week | 0.91 (0.84, 0.98)^*^ | 0.95 (0.83, 1.08) | 0.90 (0.83, 0.97)^**^ | 1.10 (0.82, 1.47) | 1.01 (0.85, 1.19) | 0.83 (0.65, 1.06) | 0.91 (0.72, 1.14) | 0.81 (0.63, 1.04) |  |
| 2-4 times a week | 0.96 (0.88, 1.04) | 0.88 (0.76, 1.02) | 0.87 (0.80, 0.95)^**^ | 1.42 (1.06, 1.90)^*^ | 0.93 (0.77, 1.13) | 0.81 (0.61, 1.06) | 0.93 (0.72, 1.21) | 0.95 (0.72, 1.25) |  |
| Daily or almost daily | 0.88 (0.83, 0.93)^***^ | 0.78 (0.70, 0.85)^***^ | 0.86 (0.81, 0.90)^***^ | 0.92 (0.75, 1.14) | 0.94 (0.83, 1.06) | 0.78 (0.65, 0.94)^**^ | 1.23 (1.05, 1.45)^*^ | 1.06 (0.89, 1.27) |  |
| Missing | 0.94 (0.82, 1.08) | 1.07 (0.87, 1.32) | 0.88 (0.77, 1.00) | 1.26 (0.81, 1.98) | 0.71 (0.51, 0.98)^*^ | 1.26 (0.88, 1.81) | 1.14 (0.79, 1.64) | 1.06 (0.73, 1.54) |  |
| Often feeling lonely |  |  |  |  |  |  |  |  |  |
| No | Ref. | Ref. | Ref. | Ref. | Ref. | Ref. | Ref. | Ref. |  |
| Yes | 1.27 (1.21, 1.34)^***^ | 1.27 (1.16, 1.39)^***^ | 1.19 (1.13, 1.26)^***^ | 1.48 (1.24, 1.77)^***^ | 1.16 (1.04, 1.30)^**^ | 0.99 (0.83, 1.18) | 0.83 (0.71, 0.98)^*^ | 0.98 (0.83, 1.16) |  |
| Missing | 1.16 (0.96, 1.41) | 1.25 (0.90, 1.74) | 1.09 (0.90, 1.33) | 1.78 (0.95, 3.33) | 1.32 (0.87, 2.01) | 0.78 (0.39, 1.57) | 0.71 (0.39, 1.29) | 1.29 (0.69, 2.43) |  |
| Friend/family visits |  |  |  |  |  |  |  |  |  |
| Never or almost never | Ref. | Ref. | Ref. | Ref. | Ref. | Ref. | Ref. | Ref. |  |
| Once every 3 months | 0.66 (0.55, 0.78)^***^ | 0.84 (0.62, 1.13) | 0.80 (0.69, 0.94)^**^ | 1.08 (0.64, 1.85) | 0.75 (0.55, 1.02) | 1.13 (0.63, 2.01) | 0.85 (0.53, 1.37) | 1.60 (0.95, 2.68) |  |
| Monthly | 0.59 (0.50, 0.69)^***^ | 0.70 (0.53, 0.93)^*^ | 0.67 (0.58, 0.78)^***^ | 0.80 (0.48, 1.34) | 0.95 (0.72, 1.26) | 1.42 (0.82, 2.46) | 1.16 (0.74, 1.80) | 1.57 (0.95, 2.59) |  |
| Weekly | 0.56 (0.48, 0.65)^***^ | 0.64 (0.49, 0.83)^***^ | 0.63 (0.55, 0.73)^***^ | 0.76 (0.47, 1.22) | 0.83 (0.64, 1.08) | 1.15 (0.68, 1.94) | 1.06 (0.70, 1.61) | 1.60 (1.00, 2.57) |  |
| 2-4 times a week | 0.54 (0.46, 0.63)^***^ | 0.71 (0.55, 0.93)^*^ | 0.65 (0.57, 0.75)^***^ | 0.83 (0.52, 1.33) | 0.70 (0.54, 0.91)^**^ | 1.11 (0.66, 1.86) | 1.07 (0.70, 1.61) | 1.42 (0.89, 2.27) |  |
| Daily | 0.58 (0.50, 0.68)^***^ | 0.71 (0.54, 0.93)^*^ | 0.64 (0.56, 0.75)^***^ | 0.80 (0.49, 1.30) | 0.72 (0.54, 0.94)^*^ | 1.24 (0.73, 2.12) | 1.12 (0.73, 1.71) | 1.31 (0.80, 2.13) |  |
| Missing | 0.53 (0.34, 0.82)^**^ | 0.60 (0.29, 1.26) | 0.79 (0.55, 1.14) | 1.09 (0.25, 4.68) | 0.63 (0.23, 1.72) | 7.76 (2.52, 23.89)^***^ | 6.74 (2.30, 19.76)^***^ | 0.46 (0.11, 1.99) |  |
| Weekly social activity |  |  |  |  |  |  |  |  |  |
| No | Ref. | Ref. | Ref. | Ref. | Ref. | Ref. | Ref. | Ref. |  |
| Yes | 0.99 (0.94, 1.03) | 0.85 (0.78, 0.92)^***^ | 0.84 (0.80, 0.87)^***^ | 0.90 (0.76, 1.07) | 0.78 (0.71, 0.87)^***^ | 0.95 (0.82, 1.11) | 0.90 (0.80, 1.02) | 1.08 (0.93, 1.25) |  |
| Missing | 1.17 (0.74, 1.84) | 1.24 (0.62, 2.49) | 1.36 (0.91, 2.03) | 1.75 (0.54, 5.67) | 0.97 (0.36, 2.63) | 0.19 (0.03, 1.40) | 0.25 (0.06, 1.01) | 2.00 (0.63, 6.31) |  |
| Living alone |  |  |  |  |  |  |  |  |  |
| No | Ref. | Ref. | Ref. | Ref. | Ref. | Ref. | Ref. | Ref. |  |
| Yes | 1.36 (1.30, 1.42)^***^ | 1.13 (1.04, 1.24)^**^ | 1.29 (1.23, 1.35)^***^ | 1.30 (1.10, 1.54)^**^ | 1.25 (1.13, 1.38)^***^ | 1.11 (0.94, 1.30) | 0.92 (0.79, 1.07) | 0.90 (0.77, 1.05) |  |
| Missing | 1.60 (1.34, 1.90)^***^ | 1.30 (0.94, 1.79) | 1.42 (1.19, 1.70)^***^ | 1.65 (0.97, 2.82) | 1.67 (1.22, 2.28)^**^ | 0.78 (0.40, 1.51) | 0.83 (0.48, 1.44) | 0.59 (0.30, 1.14) |  |
|  | **Sleep Score by transition in non-working individuals (n=101 782)** | | | | | | | | |
|  | **Healthy to fall (n=10 131)^a^** | **Healthy to dementia (n=3230)^b^** | **Healthy to death (n=10 792)^a^** | **Fall to fall & dementia (n=687)^b^** | **Fall to death (n=1985)^a^** | **Dementia to fall & dementia (n=903)^b^** | **Dementia to death (n=1223)^b^** | **Fall & dementia to death (n=896)^b^** |  |
|  | **HRs (95% CI)** | **HRs (95% CI)** | **HRs (95% CI)** | **HRs (95% CI)** | **HRs (95% CI)** | **HRs (95% CI)** | **HRs (95% CI)** | **HRs (95% CI)** |  |
| Sleep Score | 0.94 (0.93, 0.96)^***^ | 1.02 (0.99, 1.06) | 0.95 (0.93, 0.96)^***^ | 0.97 (0.90, 1.05) | 0.95 (0.91, 0.99)^*^ | 0.97 (0.91, 1.04) | 1.03 (0.97, 1.09) | 1.08 (1.00, 1.15)^*^ |  |

^a^Model was adjusted for age, sex, education, multimorbidity, hearing, vision, handgrip strength and heavy drinking.

^b^Model was adjusted for age, sex, education, multimorbidity, hearing, vision, handgrip strength, heavy drinking, and presence of APOE4-allele

HR=hazard ratio; CI=confidence interval; PA = physical activity; MET = metabolic task equivalent.

^*^P<0.05; ^**^P<0.01 ; ^***^P<0.001

**S-Table 12. Hazard ratios (HRs) and 95% confidence intervals (CIs) for multistate transitions across health, fall, dementia, and death by activity profile using posterior probabilities**

| Hazard ratios using posterior class probabilities as continuous exposures, by transition in full sample (n=288 287) | | | | | | | | |
| --- | --- | --- | --- | --- | --- | --- | --- | --- |
|  | **Healthy to fall (n=19 753)^a^** | **Healthy to dementia (n=4407)^b^** | **Healthy to death (n=19 723)^a^** | **Fall to fall & dementia (n=903)^b^** | **Fall to death (n=3141)^a^** | **Dementia to fall & dementia (n=1146)^b^** | **Dementia to death (n=1608)^b^** | **Fall & dementia to death (n=1111)^b^** |
|  | HRs (95% CI) | HRs (95% CI) | HRs (95% CI) | HRs (95% CI) | HRs (95% CI) | HRs (95% CI) | HRs (95% CI) | HRs (95% CI) |
| Active, working profile^c^ | Ref. | Ref. | Ref. | Ref. | Ref. | Ref. | Ref. | Ref. |
| Non-active, working profile^c^ | 0.98 (0.93, 1.03) | 1.32 (1.17, 1.50)^***^ | 0.99 (0.94, 1.05) | 1.67 (1.21, 2.31)^**^ | 1.12 (0.97, 1.30) | 1.00 (0.77, 1.29) | 1.05 (0.85, 1.29) | 1.11 (0.83, 1.47) |
| Active, non-working profile^c^ | 1.02 (0.97, 1.07) | 1.06 (0.92, 1.22) | 1.04 (0.99, 1.10) | 1.40 (0.97, 2.04) | 1.10 (0.94, 1.28) | 0.99 (0.72, 1.35) | 1.04 (0.81, 1.33) | 1.06 (0.75, 1.50) |
| Non-active, non-working profile^x^ | 1.81 (1.70, 1.93)^***^ | 1.96 (1.70, 2.26)^***^ | 2.12 (1.99, 2.25)^***^ | 2.95 (2.09, 4.16)^***^ | 2.06 (1.76, 2.40)^***^ | 1.38 (1.03, 1.84)^*^ | 1.01 (0.80, 1.29) | 0.95 (0.69, 1.30) |

^a^Model was adjusted for age, sex, education, Charlson Comorbidity Index, hearing, vision, handgrip strength and heavy drinking.

^b^Model was adjusted for age, sex, education, Charlson Comorbidity Index, hearing, vision, handgrip strength, heavy drinking, and presence of APOE4-allele

^c^Hazard ratios reflect a one-unit increase in the posterior probability of belonging to each activity profile, estimated from the latent class analysis. The active, working profile posterior probability is omitted as the reference.

HR=hazard ratio; CI=confidence interval.

^*^P<0.05; ^**^P<0.01; ^***^P<0.001

**References**

1. Hu H-Y, Ma Y-H, Deng Y-T, et al. Residential greenness and risk of incident dementia: A prospective study of 375,342 participants. *Environmental Research*. 2023;216(Pt 3):114703. doi:10.1016/j.envres.2022.114703.

2. Charlson M, Szatrowski TP, Peterson J, Gold J. Validation of a combined comorbidity index. *J Clin Epidemiol*. 1994;47(11):1245-1251. doi:10.1016/0895-4356(94)90129-5.

3. Charlson ME, Pompei P, Ales KL, MacKenzie CR. A new method of classifying prognostic comorbidity in longitudinal studies: development and validation. *J Chronic Dis*. 1987;40(5):373-383. doi:10.1016/0021-9681(87)90171-8.

4. Thompson A, Pirmohamed M. Associations between occupation and heavy alcohol consumption in UK adults aged 40-69 years: a cross-sectional study using the UK Biobank. *BMC Public Health*. 2021;21(1):190. doi:10.1186/s12889-021-10208-x.

5. Handley D, Gillett AC, Bala R, Tyrrell J, Lewis CM. *Latent class growth mixture modelling of HbA1C trajectories identifies individuals at high risk of developing complications of type 2 diabetes mellitus in the UK Biobank*; 2024.

6. Tein J-Y, Coxe S, Cham H. Statistical Power to Detect the Correct Number of Classes in Latent Profile Analysis. *Struct Equ Modeling*. 2013;20(4):640-657. doi:10.1080/10705511.2013.824781.

7. Lennon H, Kelly S, Sperrin M, et al. Framework to construct and interpret latent class trajectory modelling. *BMJ Open*. 2018;8(7):e020683. doi:10.1136/bmjopen-2017-020683.
